# Micro-encapsulated pirimiphos-methyl shows high insecticidal efficacy and long residual activity against pyrethroid-resistant malaria vectors in central Côte d’Ivoire

**DOI:** 10.1186/1475-2875-13-332

**Published:** 2014-08-25

**Authors:** Emile S Tchicaya, Christian Nsanzabana, Thomas A Smith, Jennifer Donzé, Maiti Laserna de Hipsl, Yao Tano, Pie Müller, Olivier JT Briët, Jürg Utzinger, Benjamin G Koudou

**Affiliations:** Département Environnement et Santé, Centre Suisse de Recherches Scientifiques en Côte d’Ivoire, Abidjan 01, Côte d’Ivoire; Unité de Formation et de Recherche Biosciences, Université Félix Houphouët-Boigny, Abidjan 22, Côte d’Ivoire; Centre for Tropical Medicine, Nuffield Department of Clinical Medicine, University of Oxford, Oxford, OX3 7LE UK; World Wide Antimalarial Resistance Network (WWARN), Oxford, OX3 7LE UK; Department of Epidemiology and Public Health, Swiss Tropical and Public Health Institute, CH–4002 Basel, Switzerland; University of Basel, CH–4003 Basel, Switzerland; Department of Parasitology, University of Neuchâtel, CH–2000 Neuchâtel, Switzerland; Centre for Neglected Tropical Diseases, Parasitology Department, Liverpool School of Tropical Medicine, Liverpool, L3 5QA UK

**Keywords:** Malaria, Insecticide resistance, Indoor residual spraying, Organophosphates, *Anopheles gambiae*, Hut trial, Côte d’Ivoire

## Abstract

**Background:**

The wide-scale implementation of insecticide-treated nets and indoor residual spraying (IRS) has contributed to a considerable decrease of malaria morbidity and mortality in sub-Saharan Africa over the last decade. Due to increasing resistance in *Anopheles gambiae* sensu lato mosquitoes to dichlorodiphenyl trichloroethane (DDT) and pyrethroids, alternative insecticide formulations for IRS with long-lasting residual activity are required to sustain the gains obtained in most malaria-endemic countries.

**Methods:**

Three experimental capsule suspension (CS) formulations of the organophosphate pirimiphos-methyl were evaluated together with Actellic 50 EC, an emulsifiable concentrate (EC) of pirimiphos-methyl, and the pyrethroid ICON 10 CS, a lambda-cyhalothrin CS formulation, in an experimental hut trial. The formulations were tested on two types of surfaces: mud and cement. The study with a 12-month follow-up was carried out in Bouaké, central Côte d’Ivoire, where *An. gambiae* mosquitoes show high levels of resistance against pyrethroids, DDT and carbamates. Residual activity was also tested in cone bioassays with the susceptible *An. gambiae* KISUMU strain.

**Results:**

One of the CS formulations of pirimiphos-methyl, CS BM, outperformed all other formulations tested. On cement surfaces, the odds ratios of overall insecticidal effect on *An. gambiae* s.l. of pirimiphos-methyl CS BM compared to Actellic 50 EC were 1.4 (95% confidence interval (CI): 1.2–1.7) for the first three months, 5.6 (95% CI: 4.4–7.2) for the second three months, and 3.6 (95% CI: 3.0–4.4) for the last six months of follow-up. On mud surfaces, the respective odds ratios were 2.5 (95% CI: 1.9–3.3), 3.5 (95% CI: 2.7–4.5), and 1.7 (95% CI: 1.4–2.2). On cement, the residual activity of pirimiphos-methyl CS BM measured using cone tests was similar to that of lambda-cyhalothrin and for both treatments, mortality of susceptible Kisumu laboratory strain was not significantly below the World Health Organization pre-set threshold of 80% for 30 weeks after spraying. Residual activity was shorter on mud surfaces, mortality falling below 80% on both pirimiphos-methyl CS BM and lambda-cyhalothrin treated surfaces at 25 weeks post-treatment.

**Conclusion:**

CS formulations of pirimiphos-methyl are promising alternatives for IRS, as they demonstrate prolonged insecticidal effect and residual activity against malaria mosquitoes.

**Electronic supplementary material:**

The online version of this article (doi:10.1186/1475-2875-13-332) contains supplementary material, which is available to authorized users.

## Background

Long-lasting insecticidal nets (LLINs) and indoor residual spraying (IRS) are the key interventions of current malaria vector control [1–3]. While IRS acts mainly by killing blood-fed mosquitoes that may be infected with the malaria parasite and thus provides protection to the wider community, LLINs primarily provide personal protection but do also show a community effect as they kill host-seeking mosquitoes. IRS using dichlorodiphenyl trichloroethane (DDT) was the mainstay of the first global malaria eradication campaign in the 1950s and 1960s [4, 5] and is among the World Health Organization’s (WHO) recommended insecticides for IRS [6]. However, due to concerns about its safety for the environment, alternatives are being sought to replace DDT [7]. Moreover, the spread of resistance to DDT and pyrethroids across sub-Saharan Africa poses a threat to insecticide-based vector control interventions [8, 9]. Available alternatives to DDT and pyrethroids are formulations of carbamates and organophosphates. However, these formulations are short-lived. Therefore, new formulations with alternative active ingredients and prolonged activity are urgently needed. A promising strategy is the repurposing of existing insecticides not currently used in public health, together with the development of improved longer lasting formulations using micro-encapsulation. A prominent candidate resulting from this strategy is micro-encapsulated pirimiphos-methyl [10–13].

Here, results from an experimental hut trial that compared the efficacy and residual activity of a lambda-cyhalothrin capsule suspension (CS) and four pirimiphos-methyl formulations, including an emulsifiable concentrate (EC) and three different CS formulations, are reported. The study was carried out in central Côte d’Ivoire where *Anopheles* mosquitoes show high levels of resistance to DDT, pyrethroids, and carbamates [14].

## Methods

### Study site

The study was conducted in central Côte d’Ivoire at the field station of the Institute Pierre Richet in the M’Bé valley near Bouaké (geographical coordinates: 7.970241° N latitude and 5.209963° W longitude). The region is a climatic transition zone with two or four seasons depending on the year. The dry season is marked by the *harmattan*, a dry wind that blows south from the Sahara from the end of November to the middle of March. The rainy season is characterized by two rainfall maxima, one in June and another in September. The average annual precipitation is between 1,000 and 1,320 mm, and was 1,370 mm between September 2008 and August 2009 when the current study was implemented. The temperature varies little throughout the year, with averages of 26 to 28°C. The annual average relative humidity is between 75 and 90%. The hydrographic network is dense, consisting of the Bandama and N’Zi rivers. The M’Bé valley is a rice-growing area providing suitable breeding sites for anthropophilic mosquitoes. The majority of the *Anopheles gambiae* sensu lato mosquitoes found in the area are highly resistant to DDT, pyrethroids and carbamates [14, 15]. Insecticide resistance is likely through metabolic mechanisms and target-site insensitivity (i.e., L1014F *kdr*) [14].

### Experimental huts

**Figure 1:**
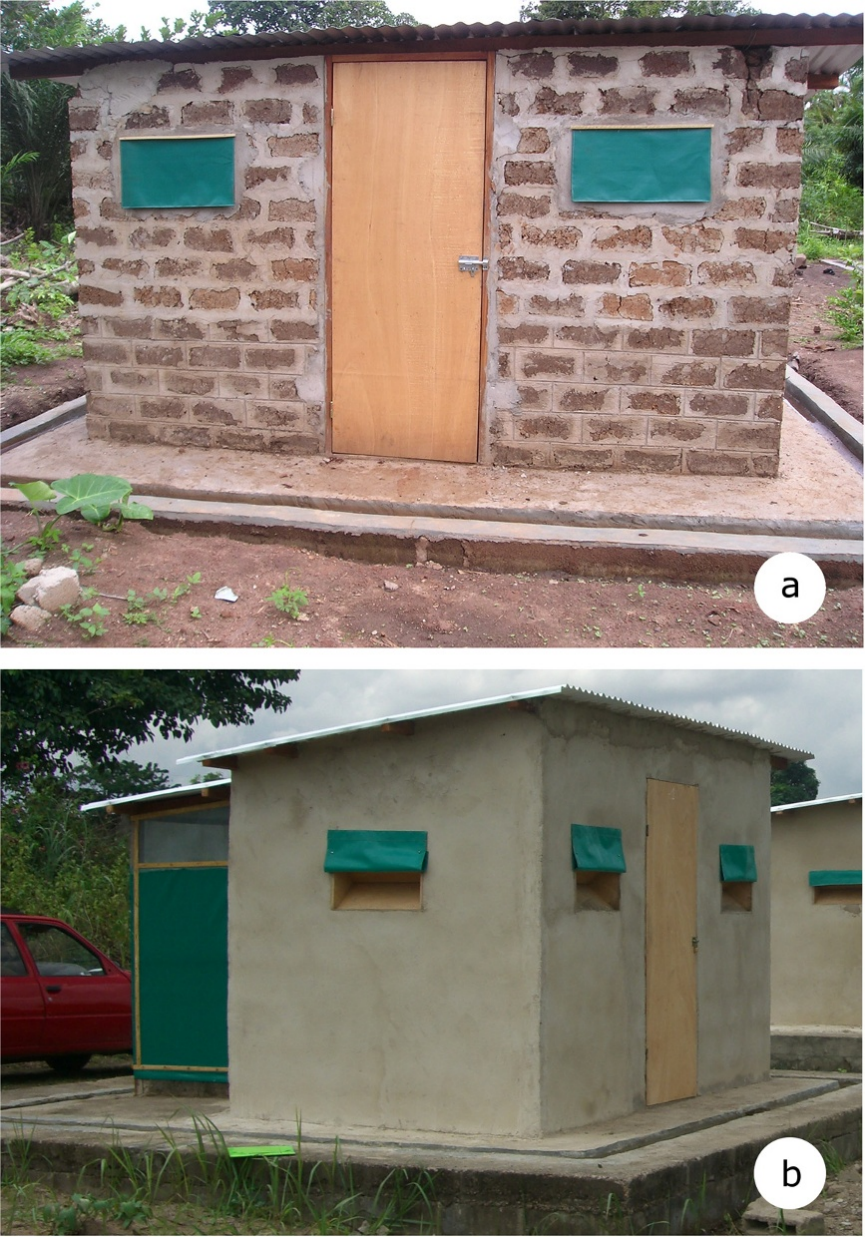
**Experimental huts used in the study located in the M’Bé site, near Bouaké in central Côte d’Ivoire.** The experimental huts comprised a sleeping room and a veranda. The walls of the room were either made of concrete bricks coated with cement **(a)** or wood structures coated in dry mud – locally called *banco* **(b)**.

Twenty-four typical West African-style experimental huts [6, 16] were built. The huts comprised of a sleeping room, into which wild mosquitoes could enter through window slits that limited their escape, and a veranda trap, into which mosquitoes could enter from the sleeping room. For 12 huts, the walls of the room were made of concrete bricks coated with cement, and for the other 12 huts, the walls were wood structures coated in dry mud (locally called *banco*) reflecting local housing construction [17, 18]. These two wall types have different porosity and alkaline characteristics [12, 19, 20]. The huts were covered with corrugated iron roofs and the ceiling consisted of polyethylene sheeting covered by palm thatch (Figure 1).

### Insecticide treatments

The insecticide formulations were produced and provided by Syngenta Crop Protection AG and included pirimiphos-methyl 50% EC (Actellic 50 EC), pirimiphos-methyl AA 30% CS, pirimiphos-methyl B 30% CS, pirimiphos-methyl BM 30% CS, and lambda-cyhalothrin 10% CS (ICON 10 CS). The formulations of pirimiphos-methyl differed in the type of solvent used to dissolve the active ingredient and in the cross-linking of the polymer capsule.

All pirimiphos-methyl treatments were applied at a target application rate of 1 g/m^2^. Lambda-cyhalothrin CS, included as a positive control, was applied at a target application rate of 0.025 g/m^2^, according to the WHO Pesticide Evaluation Scheme (WHOPES) recommendations [21]. For each treatment arm (formulation combined with either mud or cement wall surface), two huts were sprayed. An additional four huts (two mud and two cement walled huts) served as negative controls, making a total of 24 huts.

The huts were randomly allocated to insecticide treatments and their wall surfaces and palm thatch ceilings sprayed at an application rate of 35 ml/m^2^ with an aqueous solution of the aforementioned insecticides using a Hudson X-pert compression sprayer (HD Hudson Manufacturing Company; Chicago, USA) without a control valve. Spraying was carried out by a single, trained male sprayer in September 2008.

### Experimental procedure

The experimental procedure followed WHOPES guidelines for testing mosquito adulticides for IRS and treatment of mosquito nets [21]. On trapping nights, adult male sleepers identified from the communities entered the experimental huts at 18:00 hours and remained inside until 06:00 hours the next morning. The sleepers were rotated between huts on subsequent trapping nights. Trapping nights were scheduled on nights 1, 3, 5, 8, 10, 12, 15 and so on following this pattern. The follow-up duration was 12 months.

In order to measure the insecticidal efficacy of the IRS treatments, every morning at 06:00 hours, mosquitoes were collected from the rooms and the verandas and transferred to an insectary in Bouaké where the mosquitoes were scored as either dead or alive, and blood fed or unfed. Survivors were placed in small plastic cups and provided with 10% honey solution, and mortality was recorded after a 24-hour holding period.

All mosquitoes caught were identified to genus (and where possible to species) level using readily available morphological identification keys [22]. A subsample of specimens identified as *An. gambiae* s.l. was further determined using the diagnostic polymerase chain reaction (PCR) developed by Santolamezza and colleagues [23]. Template deoxyribonucleic acid (DNA) was extracted from specimens dried over silica gel following the Livak protocol [24]. Sleepers and investigators who analysed the mosquitoes in the laboratory were blinded to the identity of the treatment used in each hut, minimizing potential biases.

### Insecticide susceptibility status

In June 2009, field collected anopheline larvae were reared to adults, and *An. gambiae* s.l. were exposed for one hour to 4% DDT, 0.75% permethrin and 0.05% deltamethrin in WHO susceptibility tests [21].

### Residual activity

The residual activity of the IRS treatments was monitored on weeks 1, 5, 10, 15, 20, 25, 30, 35, 39, 42, 45, and 49 post-treatment using WHO plastic cones placed on treated surfaces, exposing female mosquitoes for 30 min and recording 24-hour mortality, according to WHOPES guidelines [21]. Replicate arms were tested on alternate testing rounds: e.g., one cement hut with Actellic 50 EC was tested on weeks 1, 10, 20, 30, 39 and 45 and the other cement hut with Actellic 50 EC was tested on weeks 5, 15, 25, 35, 42 and 49. Assays were run using susceptible KISUMU *An. gambiae* sensu stricto mosquitoes. In the insectary, the larvae were fed with Mikromin fish food (Tetra; Melle, Germany) and reared to imagines and used in the tests two to five days post eclosure from the pupae.

### Sleepers’ perception of insecticide formulations

Sleepers were interviewed by social scientists using a pre-tested questionnaire about their perception of insecticide formulations, characteristics of the odour and whether the product had an influence on their sleep quality. Questionnaires were filled in twice daily, once before sleepers entered the hut and then again the next morning after mosquito collection was completed.

### Ethical considerations

This study received ethical approval from the Ministry of Health in Côte d’Ivoire through the national malaria control programme. At the study start, the national ethics committee for research in Côte d’Ivoire was not yet functional. The study was approved by the institutional review boards. As most sleepers were illiterate, informed consent was received orally. Prior to enrolment in the study, it was ensured that all sleepers were vaccinated against yellow fever. Sleepers were medically supervised throughout the study and six months after. Suspected malaria episodes (e.g., fever and headache) were treated with an artesunate-amodiaquine combination. The young adult male sleepers were identified in the community and they received a small financial compensation for their participation. They had the right to withdraw from the study at any time without further obligations.

### Statistical analysis

Data were double-entered and cross-checked using EpiInfo version 6.4 (Centers for Disease Control and Prevention; Atlanta, USA). The mosquito data from the experimental huts were summarized per treatment arm in terms of crude rates of standard primary outcomes (crude hut entry rate, crude exit rate, crude feeding rate and crude mortality rate) for three time periods: period 1: first three months, corresponding to 40 trapping nights; period 2: second three months, corresponding to 39 trapping nights; and period 3: last six months, corresponding to 77 trapping nights.

A series of Bayesian statistical models was used to estimate the effect of the insecticide formulations on deterrence from hut entry, induced exophily, feeding inhibition, killing efficacy, personal protection, and overall insecticidal effect [25]. This was done separately for each formulation, wall type, and time period. Details of the definitions of these efficacy measures and how they were calculated are given in Additional file 1. The Bayesian approach directly provided both point and interval estimates of the insecticide efficacy measures over time, appropriately allowing for day-to-day fluctuations in mosquito density and background mortality in controls. Since it is reasonable to presume that the insecticides’ efficacies decay over time (see Additional file 2), and that the size of the host-seeking mosquito populations fluctuates strongly over the year (see Additional file 3), failure to allow for day-to-day variation in the mosquito population could potentially lead to strong biases (e.g., if the mosquito density peaked towards the end of the period of interest, due to the decayed insecticide, this would underestimate the efficacy estimate for that period). Prior distributions were defined that constrained these estimates to be between zero and unity and comparison between parameters was made by comparing the 95% credible intervals. A lack of overlap in credible intervals indicates that the differences between the effects of the insecticide formulations are unlikely to be due to chance. Summaries of experimental hut data, crude analysis and Bayesian analysis are provided as supplementary information (see Additional files 1, 2, 3 and 4). Descriptive statistics, Bayesian statistical models, and graphs were generated in the statistical software R version 2.14.1 [26].

## Results

### Mosquito species and abundance

From September 2008 to August 2009, a total of 77,948 mosquitoes were collected during 7,488 man-night catches by the young adult sleepers across all 24 experimental huts (Table 1). *Anopheles gambiae* s.l. was the predominant mosquito taxon (63.9%). Molecular analysis showed that from 120 analysed *An. gambiae* s.l. specimens 89% were *An. gambiae* s.s. The remaining *An. gambiae* s.l. specimens were *Anopheles coluzzii* (11%). Other anopheline species collected were *Anopheles funestus* (2.7%) and other not further identified *Anopheles* species (3.3%). The remaining 30.2% of the mosquitoes caught were *Mansonia africana*, *Mansonia uniformis*, *Culex* spp., or *Aedes* spp.

**Table 1:** **Number of mosquitoes collected, stratified by taxa and treatment arm over the 12-month study period (September 2008 to August 2009) in experimental huts at the M’Bé station, near Bouaké in central Côte d’Ivoire**

Species	Untreated (negative control)		Lambda-cyhalothrin ICON 10 CS (positive control)		Pirimiphos-methyl								Total (%)
					Actellic 50 EC		CS AA		CS B		CS BM		
	Cement	Mud	Cement	Mud	Cement	Mud	Cement	Mud	Cement	Mud	Cement	Mud	
*An. gambiae* s.l.	4,775	5,737	4,969	5,941	3,035	3,601	2,826	4,446	2,407	3,871	3,889	4,278	49,775 (63.9%)
*An. funestus*	324	300	132	170	116	145	100	223	78	170	114	204	2,076 (2.7%)
Other *Anopheles*	177	415	238	215	159	257	188	228	103	180	222	204	2,586 (3.3%)
Other genera (*Mansonia*, *Culex*, *Aedes*)	1,628	3,869	959	1,183	1,697	2,297	1,229	2,288	1,276	2,546	1,672	2,867	23,511 (30.2%)
Total	6,904	10,321	6,298	7,509	5,007	6,300	4,343	7,185	3,864	6,767	5,897	7,553	77,948

### Effects of insecticides on mosquitoes

**Figure 2:**
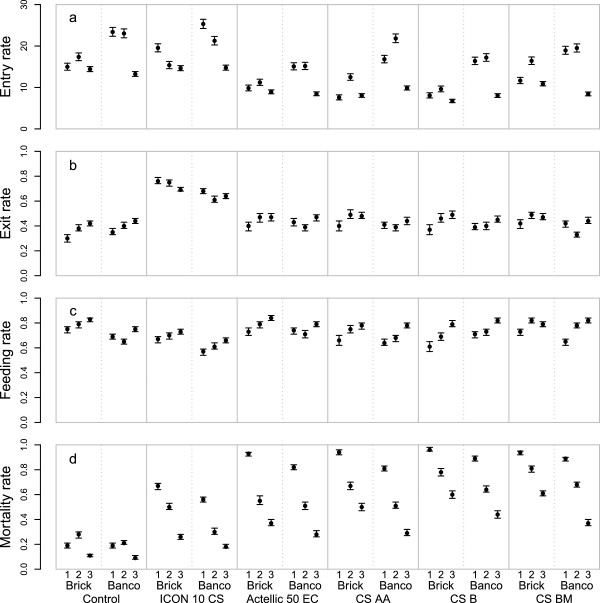
**Crude results from experimental hut trials of pirimiphos-methyl and lambda-cyhalothrin on** ***Anopheles gambiae*** **s.l.** Entry rate, number of mosquitoes per hut per night; exit rate, proportion of mosquitoes found in the veranda trap out of mosquitoes that entered; feeding rate, proportion of mosquitoes that were blood fed out of mosquitoes that entered; mortality rate, proportion of mosquitoes that were found dead or died after 24 hours post-collection out of mosquitoes that entered. The first horizontal axis labels refer to the period after spraying, with period 1 (months 1–3, corresponding to 40 trapping nights), period 2 (months 4–6, corresponding to 39 trapping nights), and period 3 (months 7–12, corresponding to 77 trapping nights). The second horizontal axis labels refer to the material of the walls of the huts where ‘brick’ refers to walls from concrete bricks coated with cement and where *banco* refers to wood structures coated in dry mud. The third (bottom) horizontal axis labels refer to experiment arms: a control, a lambda cyhalothrin (ICON 10 CS) and four different formulations of pirimiphos-methyl (Actellic 50 EC, CS AA, CS B, and CS BM).

Crude rates of standard primary outcomes for both huts per treatment arm pooled are depicted graphically in Figure 2. The entry rates (Figure 2a), the proportion that exited (Figure 2b), the proportion found to be fed (Figure 2c), and the proportion killed (Figure 2d) all varied considerably between time periods. Crude rates per hut are depicted graphically as supplementary information in Additional files 5, 6, 7, and 8 for *An. gambiae* s.l., *An. funestus*, other anophelines and other genera, respectively. Of the primary outcomes, the entry rate showed the most variation between huts of the same treatment arm.

**Figure 3:**
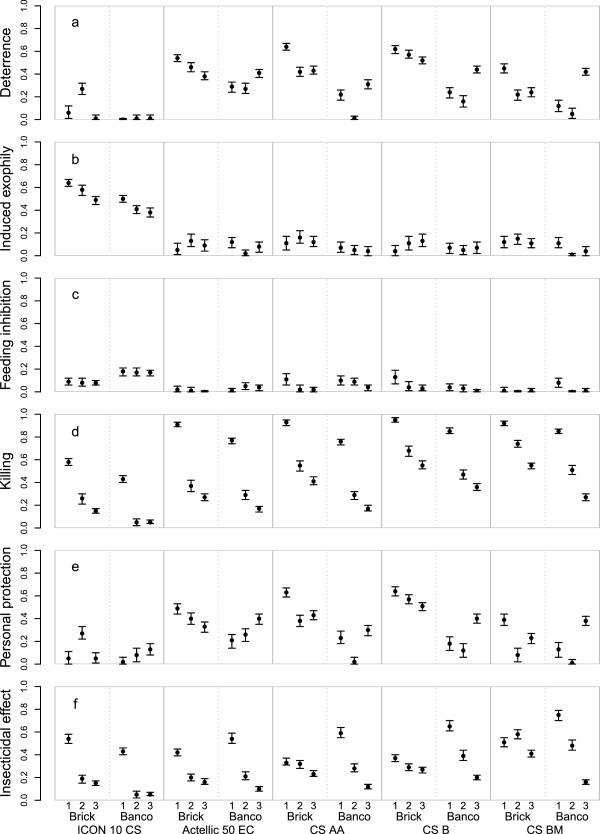
**Summary measures of the effects of pirimiphos-methyl and lambda-cyhalothrin on** ***Anopheles gambiae*** **s.l.** Summary measures are **a** deterrence from hut entry (see Additional file 1); **b** induced exophily (denoted ‘repellence’ in Additional file 1); **c** feeding inhibition (see Additional file 1); **d** killing effect (see Additional file 1); **e** personal protection, which combines effects of feeding inhibition and deterrence from hut entry [25]; and **f** overall insecticidal effect, which combines killing effect and deterrence from hut entry, adjusting for killing in control huts [25]. Points indicated posterior means and error bars indicate 95% credible intervals. The first horizontal axis labels refer to the period after spraying, with period 1 (months 1–3, corresponding to 40 trapping nights), period 2 (months 4–6, corresponding to 39 trapping nights), and period 3 (months 7–12, corresponding to 77 trapping nights). The second horizontal axis labels refer to the material of the walls of the huts where ‘brick’ refers to walls from concrete bricks coated with cement and where *banco* refers to wood structures coated in dry mud. The third (bottom) horizontal axis labels refer to the insecticide and formulation: a lambda-cyhalothrin (ICON_10CS), and four different formulations of pirimiphos-methyl (Actellic 50 EC, CS AA, CS B, and CS BM).

Effects of insecticide expressed in derived summary measures (deterrence from hut entering, induced exophily, blood feeding inhibition, killing, personal protection and insecticidal effect) on *An. gambiae s.l.* are shown in Figure 3. For *An. funestus* and other *Anopheles*, similar figures are available in Additional files 9 and 10, respectively.

The deterrent effects of lambda-cyhalothrin and pirimiphos-methyl on hut entry of *An. gambiae* s.l. are shown in Figure 3a. Whereas lambda-cyhalothrin deterrence was very low and often not significantly different from zero, deterrence was much higher with pirimiphos-methyl, preventing up to 60% of *An. gambiae* s.l. mosquitoes from entering the huts.

Induced exophily showed an inverse relationship with insecticide type as compared to deterrence. Lambda-cyhalothrin induced up to 60% of mosquitoes that entered to exit. In contrast, induced exophily with pirimiphos-methyl was very low and often not significantly different from zero (Figure 3b).

Neither pirimiphos-methyl nor lambda-cyhalothrin prevented many of the mosquitoes that entered from feeding. Lambda-cyhalothrin prevented 5–21% of entered *An. gambiae s.l.* from feeding almost consistently during the year, whereas pirimiphos-methyl only prevented 12% of entered *An. gambiae* s.l. from feeding in the fisrt three months (Figure 3c). However, pirimiphos-methyl showed the strongest lethal effects, with killing of entered *An. gambiae* s.l. being approximately double that of lambda-cyhalothrin (Figure 3d).

The killing effect in cement huts sprayed with pirimiphos-methyl CS BM was above the WHO pre-set threshold of 80% for the first three months (92%, 95% confidence interval (CI): 90–94%), was above 70% for the second three month period (74%, 95% CI: 71–77%), and above 50% for the last six months (55%, 95% CI: 52–57%). In mud huts, the killing effect of pirimiphos-methyl CS BM was about 50% during the first three months (51%, 95% CI: 47–55%) and about half of that during the second three month period (27%, 95% CI: 24–30%). In contrast, the killing effect was much lower for lambda-cyhalothrin, being 58% (95% CI: 55–61%), 26% (95% CI: 21–30%) and 15% (95% CI: 13–17%) in cement huts during periods 1, 2, and 3 respectively. Similarly, the effect was lower in mud huts sprayed with lambda-cyhalothrin. Overall, the decline in killing effect was faster in mud huts compared to cement huts, and varied between the different products, with pirimiphos-methyl formulations CS B and CS BM having the highest effect for the longest period in both hut types.

Results for personal protection against *An. gambiae* s.s. bites were variable. For example, during period 1 and period 3, personal protection was greater for pirimiphos-methyl formulation CS BM than for lambda-cyhalothrin (Figure 3e), but it was inverse during period 2.

The overall insecticidal effect of CS formulations of pirimiphos-methyl on cement walls was relatively stable over time compared to on mud walls, where the effect was stronger during the first period but dropped faster in the two following periods (Figure 3f). On cement, the insecticidal effect of pirimiphos-methyl EC and of lambda-cyhalothrin dropped faster between the first and second three-month periods compared to the drop in effect of pirimiphos-methyl CS. On mud, the insecticidal effect of all insecticide formulations decayed relatively fast.

**Figure 4:**
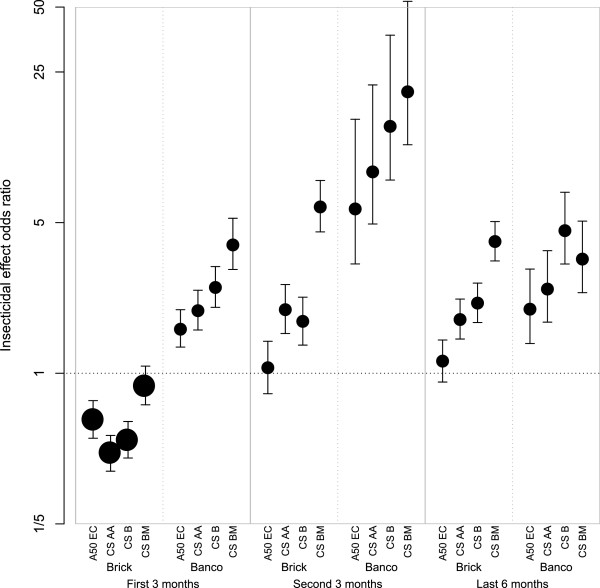
**Odds ratio of overall insecticidal effect of pirimiphos-methyl relative to lambda-cyhalothrin on** ***Anopheles gambiae*** **s.l.** Points indicate posterior means and error bars indicate 95% credible intervals. The first horizontal axis labels refer to the four different formulations of pirimiphos-methyl (Actellic 50 EC, CS AA, CS B and CS BM). The second horizontal axis labels refer to the material of the walls of the huts where ‘brick’ refers to walls from concrete bricks coated with cement and where *banco* refers to wood structures coated in dry mud. The third (bottom) horizontal axis labels refer to the period after spraying, with period 1 (months 1–3, corresponding to 40 trapping nights), period 2 (months 4–6, corresponding to 39 trapping nights), and period 3 (months 7–12, corresponding to 77 trapping nights).

Figure 4 provides a visualization of the overall insecticidal effect on *An. gambiae* s.l. of pirimiphos-methyl formulations relative to lambda-cyhalothrin. For the overall insecticidal effect on *An. gambiae* s.l., with the exception of CS BM, pirimiphos-methyl was significantly inferior to lambda-cyhalothrin during the first three months on cement surfaces (Figure 4). However, on mud surfaces, pirimiphos-methyl was significantly superior to lambda-cyhalothrin. For the other periods of follow-up on cement, the overall insecticidal effect of Actellic 50 EC was not significantly different from the effect of lambda-cyhalothrin. However, on mud surfaces, it did outperform lambda-cyhalothrin (Figure 4). For the second trimester and last six months, all CS formulations of pirimiphos-methyl outperformed lambda-cyhalothrin (Figure 4). When ranking the different formulations the order is: Actellic 50 EC (least good), CS AA, CS B, and CS BM (best). The highest odds ratio relative to lambda-cyhalothrin was estimated for CS BM on mud for the second three-month period after application, with an odds ratio of 18.4 (95% CI: 10.6–48.4). For *An. funestus* and other *Anopheles*, similar figures are available in Additional files 11 and 12, respectively.

**Figure 5:**
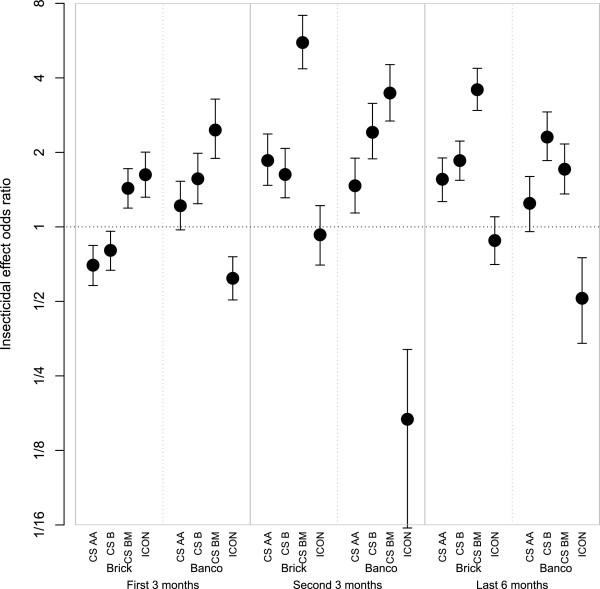
**Odds ratio of overall insecticidal effect of pirimiphos-methyl CS and lambda-cyhalothrin relative to pirimiphos-methyl EC (Actellic 50 EC) on** ***Anopheles gambiae*** **s.l.** Points indicate posterior means and error bars indicate 95% credible intervals. The first horizontal axis labels refer to the three different CS formulations of pirimiphos-methyl (CS AA, CS B and CS BM) and lambda-cyhalothrin (ICON 10 CS). Legend further as in Figure 4.

Figure 5 provides a comparison of the overall insecticidal effect on *An. gambiae s.l.* of the CS formulations of pirimiphos-methyl compared to the EC formulation of pirimiphos-methyl. During the first three months on cement surfaces, formulations of CS AA and CS B were inferior to the EC formulation on cement, with odds ratios being slightly but significantly below one. For the first three months and also for the last six months, on mud surfaces, the median odds ratio for CS AA was higher than one but this was not significant as the 95% CI included one. For all other comparisons, the odds ratios for CS formulations were significantly higher than one. Formulation CS BM had generally the highest odds ratios, except on mud surfaces for the last six months, where formulation CS B was somewhat higher.

### Insecticide susceptibility status

The results of the WHO susceptibility test experiment are given in Table 2. Twenty-four hours post-exposure, mortality was 0, 4.5, 10.6 and 44.6% in control, DDT, permethrin and deltamethrin arms, respectively.

**Table 2:** **Knock down and mortality of wild** ***An. gambiae*** **s.l. in WHO susceptibility tests**

Treatment	Knocked down (%)	Mortality (%)	n
Control	0	0	50
DDT 4%	3.0	4.5	132
Deltamethrin 0.05%	56.1	44.6	139
Permethrin 0.75%	0.6	10.6	160

Legend: n = number tested.

### Residual activity

**Figure 6:**
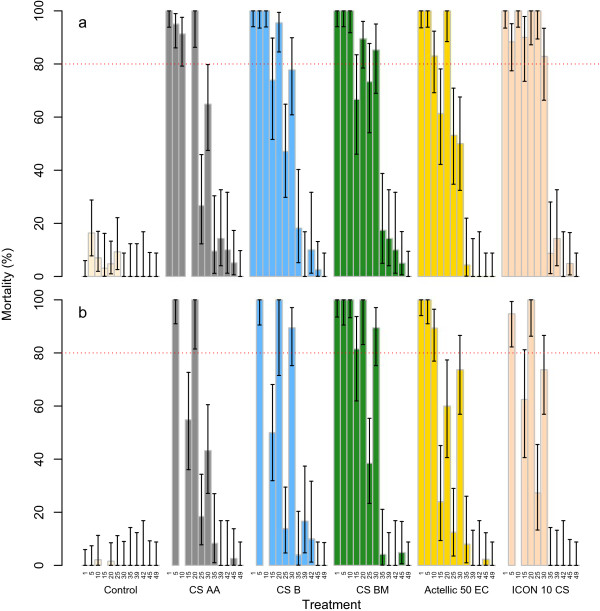
**Residual activity of insecticide formulations against susceptible** ***Anopheles gambiae.*** Mortality in WHO cone tests after 24 hours of *An. gambiae* of the KISUMU strain **a** on cement surfaces, and **b** on mud surfaces. Error bars show 95% confidence intervals. The red dotted line shows the WHO threshold of 80%. The first horizontal axis labels refer to the week of testing post-treatment.

Residual activity of the insecticide formulations against the susceptible KISUMU strain, as measured by the WHO cone bioassays, did not vary much with surface, with 95% CIs mostly overlapping for matching test rounds and insecticide formulations (Figure 6). However, the residual activity was longer on cement surfaces. The observed mortality of KISUMU on cement surfaces treated with lambda-cyhalothrin or the CS BM formulation of pirimiphos-methyl was not significantly below the WHO 80% threshold for the first 30 weeks after application. For pirimiphos-methyl CS AA and CS B, this was for the first 20 weeks, and for Actellic 50 EC this was 10 weeks. On mud surfaces, the residual effect was shorter: for pirimiphos-methyl CS BM and lambda-cyhalothrin, mortality was not significantly below 80% for the first 20 weeks. For mud surfaces treated with CS AA, CS B, and EC formulations of pirimiphos-methyl, mortality was significantly below 80% 15 weeks after application.

### Sleepers’ perception of insecticide formulations

Differences were found in the perceived strength and odour of the insecticides according to the formulations of pirimiphos-methyl and depending on the wall type of the experimental huts (see Additional file 13). On huts constructed from cement bricks, formulations CS B and CS AA were considered the least dangerous, but somewhat less efficient and had an odour which was less pleasant. On mud walled huts, formulation CS BM was considered to have the strongest odour and efficiency, but was perceived somewhat more dangerous than the other products.

## Discussion

Currently four classes of insecticides targeting adult mosquitoes are endorsed by WHO for use in IRS: pyrethroids, organochlorines, carbamates and organophosphates [6]. Pyrethroids exhibit low mammalian toxicity in their application and a rapid knock-down effect [27] and the formulations for IRS are relatively long-lasting (at least three months) [28]. DDT, the only available organochlorine for IRS, shows rapid knock-down effect and relative longevity but due to resistance and environmental concerns, its use in vector control programmes is declining as alternative insecticides with better target product profiles are becoming available [28, 29]. Bendiocarb, representing the carbamates, is also used for IRS, is highly effective and shows little excito-repellency. Yet, bendiocarb has a short residual life and is not a favourable option in areas of long or perennial malaria transmission as multiple applications would be required to cover the whole season. The last class is that of the organophosphates to which pirimiphos-methyl belongs. The organophosphates are similar to the carbamates in their mode of action and are generally also rather short-lived (as found for Actellic 50 EC in this study). While bendiocarb (a carbamate) and organophosphates have the downside of showing a short residual life, they may become viable alternatives in areas with a short transmission season as resistance to pyrethroids – and cross-resistance to DDT – becomes widespread in *Anopheles* populations threatening the sustenance of gains made in the control of malaria with pyrethroid-based LLINs and IRS [9]. Micro-encapsulation of pirimiphos-methyl makes this active ingredient an alternative in areas with pyrethroid resistance where the malaria transmission season is long.

On cement walls, residual activity of both lambda-cyhalothrin and pirimiphos-methyl CS lasted for up to 30 weeks as compared to 10 weeks with the EC formulation, corroborating recent observations in Tanzania [10, 12]. On mud walls, however, residual activity of both lambda-cyhalothrin and pirimiphos-methyl was shorter, suggesting a strong influence of the substrate to which it is applied. It has been documented before that the persistence of an insecticide depends on a number of factors, including the type of the surface and formulation type. On traditional mud walls, the approximate duration of the residual effect of two organophosphate insecticides (i.e., malathion and fenitrothion) is three months^a^ and of DDT is six months [30]. Malathion sprayed on wood may last more than three months, whereas on some mud surfaces it may last for only three weeks [30]. Mud surfaces generally absorb some of the applied insecticide and certain types of mud may also break down insecticides chemically [19, 20].

Towards the end of the experimental hut trial, in June 2009, wild mosquitoes in M’Bé showed 10.6 and 44.6% mortality with 0.75% permethrin and 0.05% deltamethrin, respectively. No contemporary data on mortality with lambda-cyhalothrin and pirimiphos-methyl are available but permethrin deltamethrin and lambda-cyhalothrin are all pyrethroids, so cross-resistance may be roughly comparable. For instance, in Yaokoffikro, 35 km south of M’Bé, in June 2008, Koffi and colleagues [31] observed 69% and 68% mortality with 1% permethrin and 0.05% lambda-cyhalothrin, respectively. If cross-resistance in M’Bé is similar, a mortality of about 10.6% could be expected with 0.05% lambda-cyhalothrin. However, in M’Bé, in May 2012, Koffi and colleagues [14] observed 51.2%, 75.8% and 98.1% mortality with 0.75% permethrin, 0.05% deltamethrin and 1% pirimiphos-methyl, respectively. As the mortality was higher with pyrethroids in 2012 than observed in 2009, it is possible that the M’Bé *An. gambiae* population had lost some pyrethroid resistance. Since exposure of the M’Bé *An. gambiae* population to pirimiphos-methyl prior to this study was probably low and measured susceptibility in 2012 was high, the *An. gambiae* population in 2009 was probably fully susceptible to this insecticide. Potentially lower pyrethroid resistance in Yaokoffikro (68.9% mortality in 2008 with 1% permethrin), compared with M’Bé (10.6% and 51.2% mortality with 0.75% permethrin in 2009 and 2012, respectively) may explain why activity of lambda-cyhalothrin was comparable to that of pirimiphos-methyl when tested in cone tests with *An. gambiae* from Yaokoffikro (see Additional file 14).

The experimental hut results on mortality with free flying *An. gambiae* s.l. were consistent with the results from cone tests for residual activity: both effects on mortality and residual activity lasted longer on cement than on mud, and longer for pirimiphos-methyl CS BM than for Actellic 50 EC. Indeed, the lower effects on mortality of free flying *An. gambiae* s.l. of lambda-cyhalothrin, despite its persistent activity, can be attributed to pyrethroid resistance.

The positive control (lambda-cyhalothrin) also showed greater insecticidal effects on cement than on mud huts, but it killed a much lower proportion of the mosquitoes that had entered the huts than did pirimiphos-methyl, with a substantial proportion of mosquitoes diverted into the veranda. Surviving unfed mosquitoes were found in pyrethroid-treated huts, indicating that pyrethroid inhibited feeding. Such mosquitoes were infrequent in pirimiphos-methyl treated huts, where mosquitoes had high blood-feeding rates, which is in agreement with results from experimental hut trials where pirimiphos-methyl was applied to bed nets [32].

The numbers of mosquitoes entering the pirimiphos-methyl-treated huts were lower than with either control or pyrethroid-treated huts, suggesting that the latter deterred mosquitoes from entering. This apparent deterrence was the main driver of the estimated high personal protection effects of the insecticide. In general, organophosphate insecticides are considered to be non-irritant [33], so the observation of deterrency is somewhat surprising, given the low level of excito-repellency observed with pirimiphos-methyl [10] but consistent with hut studies in Benin [34]. Perhaps the design of the experimental hut may explain some of the observed variation.

As blank collections immediately prior to the study suggested little bias between huts, each treatment was randomly assigned to two huts, and data from the two huts that received the same treatment were pooled for all subsequent analyses. However, there was considerable variation between huts in numbers of mosquitoes entering (see Additional files 5, 6, 7 and 8), presumably because house entering depended on the locations of the huts relative to breeding sites and on external environmental features affecting mosquito movement. It would be recommendable for future hut trials to include more replications per arm, or to rotate sprayed panels, in order to reduce any bias in deterrence (from hut entry) estimates due to hut location. In contrast, the hut location did not appear to have had a notable effect on estimates of feeding inhibition, killing, or repellency, for which variations between huts were reduced by standardizing hut design.

The survival of mosquitoes in control huts showed temporal variation, making it essential to allow for a temporal dimension in the analysis of the insecticidal efficacy (defined as the proportion of mosquitoes killed among those that would have survived in negative control huts). The temporal fluctuations in mortality rates were also observed in the control bioassays and might be explained by the desiccating effects of the *harmattan*. An alternative to the Bayesian approach applied in the current study would have been to use generalized linear mixed model (GLMM) approaches to calculate mortality and feeding, with only crude adjustment for temporal variations in mosquito densities and insecticidal effects. This would have required plug-in estimates from the GLMMs into the formulae for the insecticide efficacy measures, leading to potentially biased estimates and CIs based on complex approximations.

Recent studies have shown that IRS applications might lack precision, calling for standardized testing guidelines [35]. During the spraying of huts, filter papers were exposed and sent overseas for the analysis of the insecticide dose. Unfortunately, due to substantial delays, accurate readings could not be obtained. Therefore, no confirmation is available that the specified insecticide target doses were obtained. Despite adhering to WHOPES guidelines and standard protocols with the spraying, these results must be interpreted with some care. However, products were diluted according to recommendations and as all the applications were made by a single operator, one could assume that any errors would be similar across treatments.

The effect of IRS on malaria transmission is primarily measured by its overall insecticidal effect [25]. In the present analyses, the overall insecticidal effect was computed conservatively assuming that neither deterrence nor repellency affects mosquito viability. Overall, taking into account estimated deterrency, repellency, blood-feeding rates and killing effects, pirimiphos-methyl provided both greater personal protection and a larger overall insecticidal effect against *An. gambiae* s.l. than did the pyrethroid control, on both tested surfaces.

## Conclusions

CS formulations of pirimiphos-methyl showed higher efficacy against pyrethroid-resistant *Anopheles* mosquitoes than lambda-cyhalothrin CS and extended life span compared to the EC formulation. The results presented here stemming from a large experimental hut trial conducted in an area of Côte d’Ivoire where malaria vectors are resistant against pyrethroids [14] confirm that pirimiphos-methyl CS is a valuable alternative IRS insecticide [12, 33]. Hence, pirimiphos-methyl CS might be considered to replace pyrethroids in areas where resistance to the latter class of insecticides is widespread or developing, which in turn might slow the development of pyrethroid resistance.

## Endnote

^a^A more recent WHO publication [36] gives duration ranges for malathion of 2–3 months, and for fenitrothion of 3–6 months, stating “It should be noted that the residual effect of insecticides can be much shorter on some surfaces, such as porous mud walls, walls covered by cement or alkaline whitewash and surfaces exposed to sunlight”.

## Electronic supplementary material

Additional file 1: **Details of statistical methods.** (PDF 24 KB)

Additional file 2: **Alternative presentation of experimental hut results.** (PDF 231 KB)

Additional file 3: **Total numbers of nightly mosquito catches in experimental huts.** (PDF 115 KB)

Additional file 4: **Summaries of data and results.** (XLS 318 KB)

Additional file 5: **Crude results from experimental hut trials of pirimiphos-methyl and lambda-cyhalothrin on** ***Anopheles gambiae*** **s.l. by hut.** (PDF 37 KB)

Additional file 6: **Crude results from experimental hut trials of pirimiphos-methyl and lambda-cyhalothrin on** ***Anopheles funestus*** **by hut.** (PDF 37 KB)

Additional file 7: **Crude results from experimental hut trials of pirimiphos-methyl and lambda-cyhalothrin on other than** ***Anopheles gambiae*** **s.l. and** ***Anopheles funestus*** **by hut.** (PDF 40 KB)

Additional file 8: **Crude results from experimental hut trials of pirimiphos-methyl and lambda-cyhalothrin on other genera by hut.** (PDF 36 KB)

Additional file 9: **Effects of pirimiphos-methyl and lambda-cyhalothrin on** ***Anopheles funestus*** **.** (PDF 40 KB)

Additional file 10: **Effects of pirimiphos-methyl and lambda-cyhalothrin on anophelines other than** ***Anopheles gambiae*** **s.l. and** ***Anopheles funestus*** **.** (PDF 41 KB)

Additional file 11: **Odds ratio of overall insecticidal effect of pirimiphos-methyl relative to lambda-cyhalothrin on** ***Anopheles funestus*** **.** (PDF 28 KB)

Additional file 12: **Odds ratio of overall insecticidal effect of pirimiphos-methyl relative to lambda-cyhalothrin on on anophelines other than** ***Anopheles gambiae*** **s.l. and** ***Anopheles funestus*** **.** (PDF 29 KB)

Additional file 13: **Perceived strengths and weakness of different insecticide formulations in experimental huts.** (PDF 15 KB)

Additional file 14: **Results of WHO cone tests with wild** ***An. gambiae*** **s.l. from Yaokoffikro.** (PDF 70 KB)

## Abbreviations

CI: Confidence interval
CS: Capsule suspension
DDT: Dichloro-diphenyl-trichloroethane
DNA: Deoxyribonucleic acid
EC: Emulsifiable concentrate
GLMM: Generalized linear mixed model
IRS: Indoor residual spraying
LLIN: Long-lasting insecticidal net
PCR: Polymerase chain reaction
WHO: World Health Organization
WHOPES: World Health Organization Pesticide Evaluation Scheme.
