# Supplementary material for: Micro-encapsulated pirimiphos-methyl shows high insecticidal efficacy and long residual activity against pyrethroid-resistant malaria vectors in central Côte d’Ivoire
Source: Malar J. 2014 Aug 25;13:332. doi: 10.1186/1475-2875-13-332 (PMC4159530; doi:10.1186/1475-2875-13-332)
Supplement: Supplementary file 1 — Additional file 1: Details of statistical methods. (PDF 24 KB) [file 12936_2014_3370_MOESM1_ESM.pdf]

Additional file 1. Details of statistical methods.

The deterrence at time *t*, $\delta(k,t)$ for treatment *k*, is defined as the proportionate reduction in the entry rate, as measured by:

$$\delta(k,t) = 1 - \frac{E(k,t)}{E(0,t)},$$

where $E(k,t)$ is the total number of female mosquitoes found in an experimental hut and veranda with treatment *k*, where $k = 0$ corresponds to control (untreated) huts at time *t*.

The repellence at time *t*, $\rho(k,t)$ for treatment *k*, is defined as the proportionate decrease in the proportion of female mosquitoes remaining in the hut, rather than in the veranda trap, as measured by:

$$\rho(k,t) = 1 - \frac{R(k,t)}{R(0,t)},$$

where $R(k,t)$ is the proportion of the female mosquitoes remaining in the hut (rather than exiting into the veranda trap) with treatment *k*. Similarly, feeding inhibition is defined as the reduction in the proportion of mosquitoes blood-fed, as measured by:

$$\phi(k,t) = 1 - \frac{F(k,t)}{F(0,t)},$$

where $F(k,t)$ is the expected proportion of female mosquitoes that are fed with treatment k and the killing effect is measured by $\omega(k,t)$, defined as:

$$\omega(k,t) = 1 - \frac{S(k,t)}{S(0,t)},$$

where $S(k,t)$ is the expected proportion of female mosquitoes that survive (among those entering the hut).

Statistical inferences about deterrence, repellence, feeding inhibition and mosquito survival were made using Bayesian hierarchical models. Specifically, $\delta(k,0)$ and β_D were estimated by assuming the observed numbers of mosquitoes in each experimental hut on each day to be Poisson distributed about the expectation defined by rearranging the equation given above, *i.e.*

$$e(h,t) \sim Poisson\left(E(k(h),t)\right),$$

where *k(h)* denotes the treatment of hut *h*. To allow for day-to-day fluctuations in the density of host seeking mosquitoes, the expected numbers of mosquitoes entering the control huts were modeled as log-normally distributed, as follows:

$$\ln\left(E(0,t)\right) \sim Normal(\mu_D, \sigma_D^2),$$

where σ_D^2 and μ_D are hyperparameters with non-informative prior distributions.
Similar models were used for estimating $R(k,t)$, $F(k,t)$, and $S(k,t)$, but since these are proportions they were formulated as logistic, rather than Poisson models. In each case the Bayesian models were fitted using WinBUGS version 1.4 software and credible intervals calculated by sampling the posterior distributions. In addition the personal protection effect of IRS was estimated by a further Poisson model analogous to the model for house entry and deterrence but where the outcome was the number of blood fed mosquitoes found in the house, rather than the total number entering.

The effect of IRS on malaria transmission is measured by the overall insecticidal effect, which is the proportion of all host seeking mosquitoes that are killed. The overall insecticidal effect was calculated as:

$$K(k,t) = \omega(k,t)\left(1-\delta(k,t)\right),$$

corresponding to the lower bound for the true overall effect, based on the assumption that deterred mosquitoes are find alternative hosts in houses without IRS and are not killed. The odds ratio measuring the effect of a given pirimiphos-methyl formulation relative to lambda-cyhalothrin (treatment 1) was defined as:

$$OR = \frac{K(k,t)\left(1-K(1,t)\right)}{K(1,t)\left(1-K(k,t)\right)}.$$
