# Supplementary material for: Micro-encapsulated pirimiphos-methyl shows high insecticidal efficacy and long residual activity against pyrethroid-resistant malaria vectors in central Côte d’Ivoire
Source: Malar J. 2014 Aug 25;13:332. doi: 10.1186/1475-2875-13-332 (PMC4159530; doi:10.1186/1475-2875-13-332)
Supplement: Supplementary file 2 — Additional file 2: Alternative presentation of experimental hut results. (PDF 231 KB) [file 12936_2014_3370_MOESM2_ESM.pdf]

Additional file 2: Alternative presentation of experimental hut results.

Table 2.1 Summary table. Summary of results for *An. gambiae* s.l., *An. funestus* and non-anopheline genera of the experimental hut trial, following the analysis and analysis used in Tables 1 – 2 in [10].

Species	Material	Treatment	Total collected	% Caught in veranda (95%CI)	% Bloodfed (95%CI)	% Mortality (95%CI)	% Corrected mortality (95%CI)
An. gambiae	Cement	Untreated	4776	38a (37-39)	80a (78-81)	18a (17-19)	-
		CS AA	2822	47b (45-49)	74b (73-76)	65b (64-67)	58 (56-60)
		CS B	2434	45b (43-47)	71bc (70-73)	75c (73-77)	70 (68-72)
		CS BM	3893	46b (45-48)	79a (77-80)	75c (74-77)	70 (68-72)
		Actellic 50 EC	3041	45b (43-47)	80a (78-81)	57d (55-59)	48 (45-50)
		ICON 10 CS	5019	73c (72-74)	70c (69-72)	44e (43-46)	33 (31-35)
	Mud	Untreated	5710	40ab (39-41)	70a (69-71)	16a (15-17)	-
		CS AA	4569	41ab (40-43)	70a (69-72)	53b (51-54)	43 (42-45)
		CS B	3893	41ab (40-43)	75b (74-76)	66c (65-68)	60 (58-61)
		CS BM	4337	39ab (38-41)	75b (73-76)	66c (65-67)	60 (58-61)
		Actellic 50 EC	3694	43b (41-44)	75b (73-76)	53b (51-55)	44 (42-46)
		ICON 10 CS	5959	65c (63-66)	62c (60-63)	34d (33-36)	22 (20-23)
An. funestus	Cement	Untreated	341	26a (22-31)	86a (82-90)	34a (29-39)	-
		CS AA	104	25a (17-34)	70bc (60-79)	88b (81-94)	83 (73-91)
		CS B	83	27a (17-37)	72bc (61-82)	83bc (73-90)	75 (62-86)
		CS BM	125	36a (28-45)	70bc (62-78)	87b (80-93)	81 (71-89)
		Actellic 50 EC	122	32a (24-41)	82ab (74-88)	72c (63-80)	58 (45-70)
		ICON 10 CS	137	64b (55-72)	55c (47-64)	75bc (67-82)	62 (51-73)
	Mud	Untreated	306	31a (26-37)	77a (72-82)	33a (28-39)	-
		CS AA	235	33a (27-39)	78a (72-83)	82b (77-87)	73 (65-80)
		CS B	183	30a (24-37)	72ab (64-78)	86b (80-91)	79 (72-87)
		CS BM	215	32a (25-38)	63b (56-70)	85b (80-90)	78 (70-85)
		Actellic 50 EC	160	24a (18-32)	74ab (66-80)	81b (74-87)	72 (62-81)
		ICON 10 CS	177	61b (53-68)	66a (58-73)	64c (56-71)	46 (34-57)
Non-anopheline Genera	Cement	Untreated	1608	59a (57-62)	59a (56-61)	9a (7-10)	-
		CS AA	1202	52b (49-55)	50b (47-53)	37b (34-39)	31 (27-34)
		CS B	1301	54ab (52-57)	46b (44-49)	38b (35-40)	32 (29-35)
		CS BM	1691	56ab (54-59)	57a (55-60)	40b (38-42)	34 (31-37)
		Actellic 50 EC	1704	58a (56-60)	61a (59-64)	32c (30-34)	25 (23-28)
		ICON 10 CS	959	49b (46-53)	16c (14-18)	62d (59-65)	58 (55-61)
	Mud	Untreated	3884	54a (53-56)	61a (60-63)	11a (10-12)	-
		CS AA	2279	54a (52-56)	67b (65-68)	34b (32-36)	26 (23-28)
		CS B	2519	59b (57-61)	74c (72-75)	32b (30-34)	23 (21-25)
		CS BM	2843	55a (53-56)	66b (64-67)	41c (39-42)	33 (31-35)
		Actellic 50 EC	2264	54a (52-56)	64ab (62-66)	32b (30-34)	24 (21-26)
		ICON 10 CS	1170	43c (40-46)	25d (22-27)	58d (55-61)	53 (50-56)

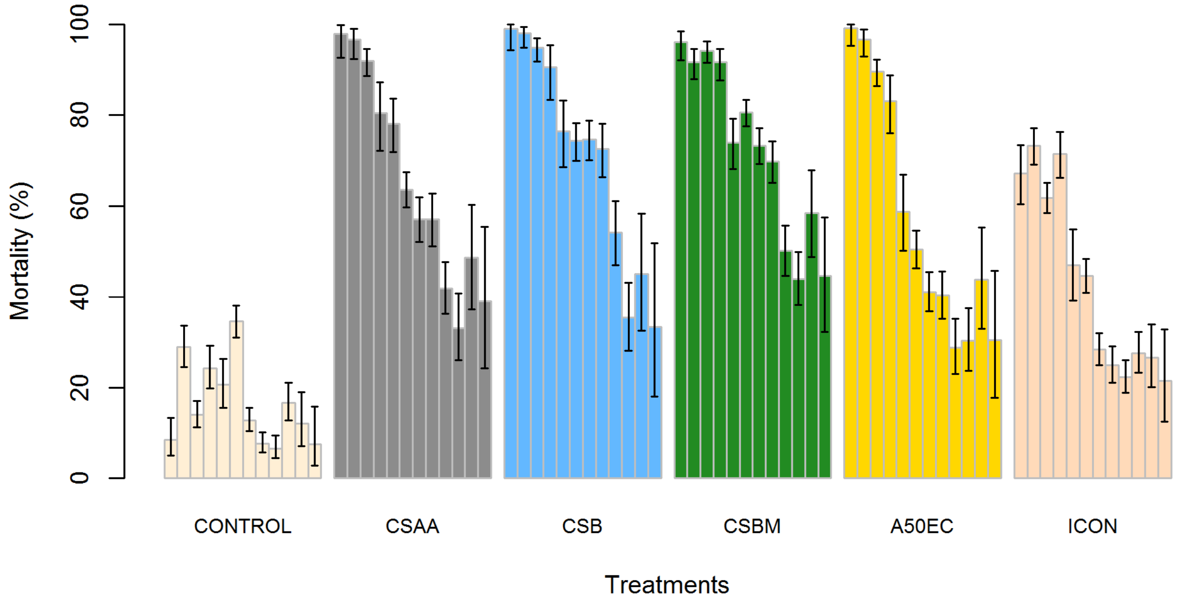

Figure 2.1. Crude mortality of *An. gambiae* s.l. in cement huts by month post treatment. The first month is on the left. Error bars show 95% confidence intervals. This presentation allows comparison of the results of this trial with those presented in [10].

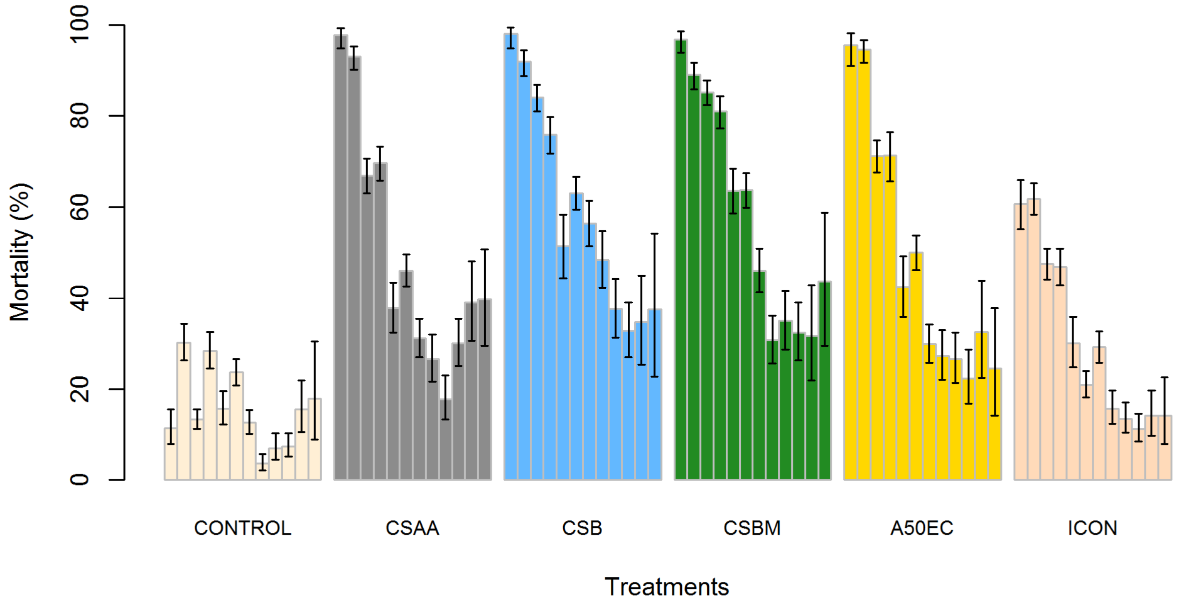

Figure 2.2. Crude mortality of *An. gambiae* s.l. in mud huts by month post treatment. See Figure 2.1 for legend.

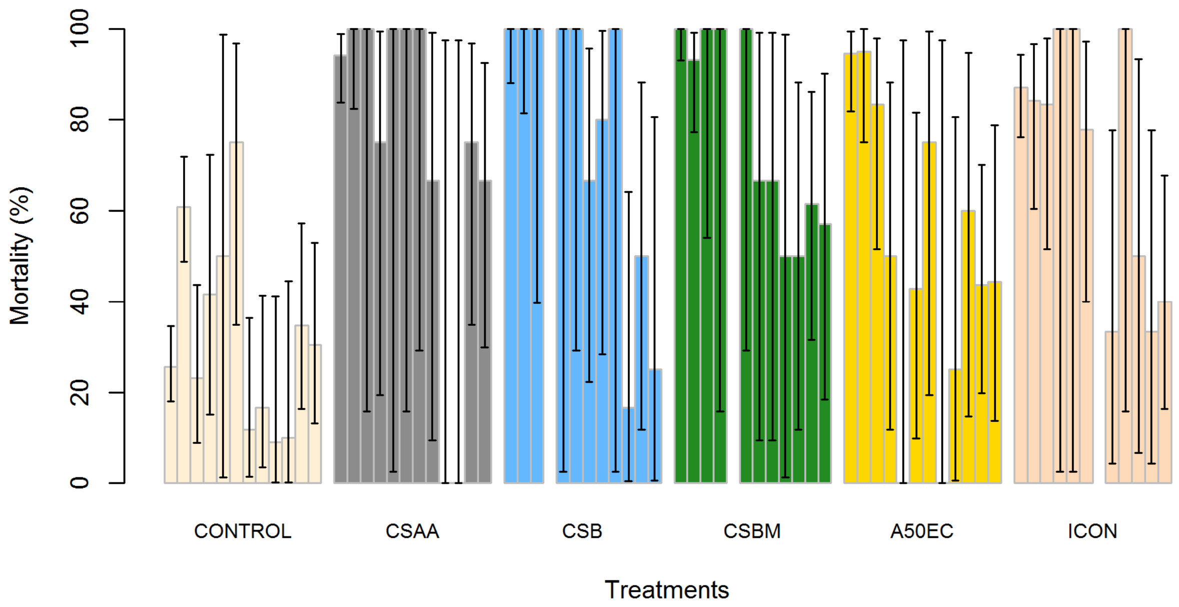

Figure 2.3. Crude mortality of *An. funestus* in cement huts by month post treatment. See Figure 2.1 for legend.

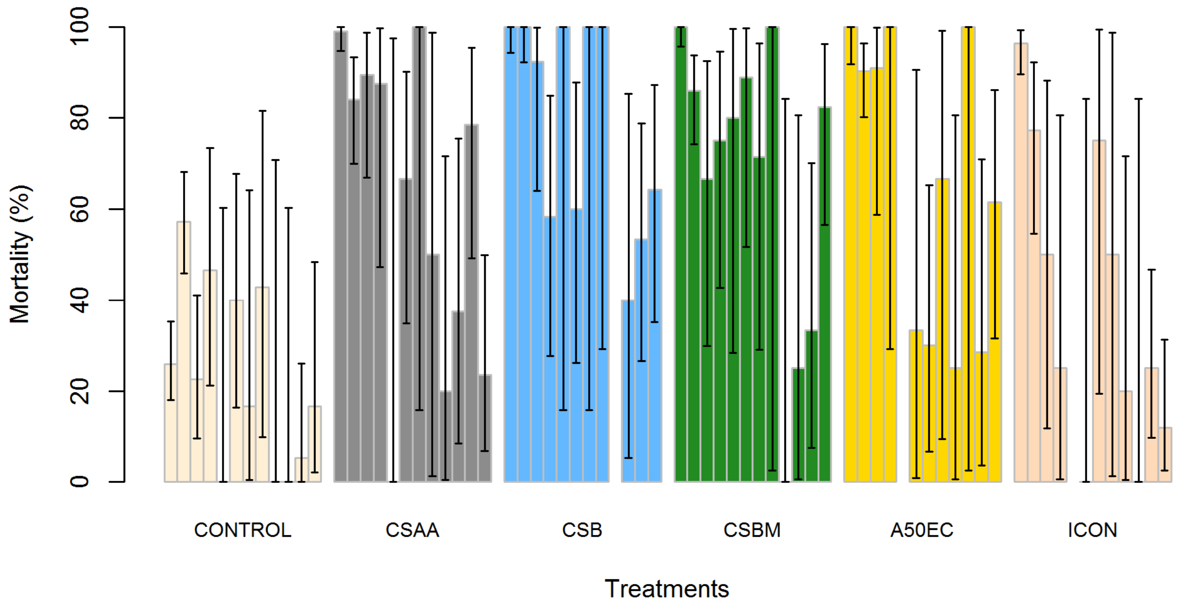

Figure 2.4. Crude mortality of *An. funestus* in mud huts by month post treatment. See Figure 2.1 for legend.

Mortality of *An. funestus* in mud huts

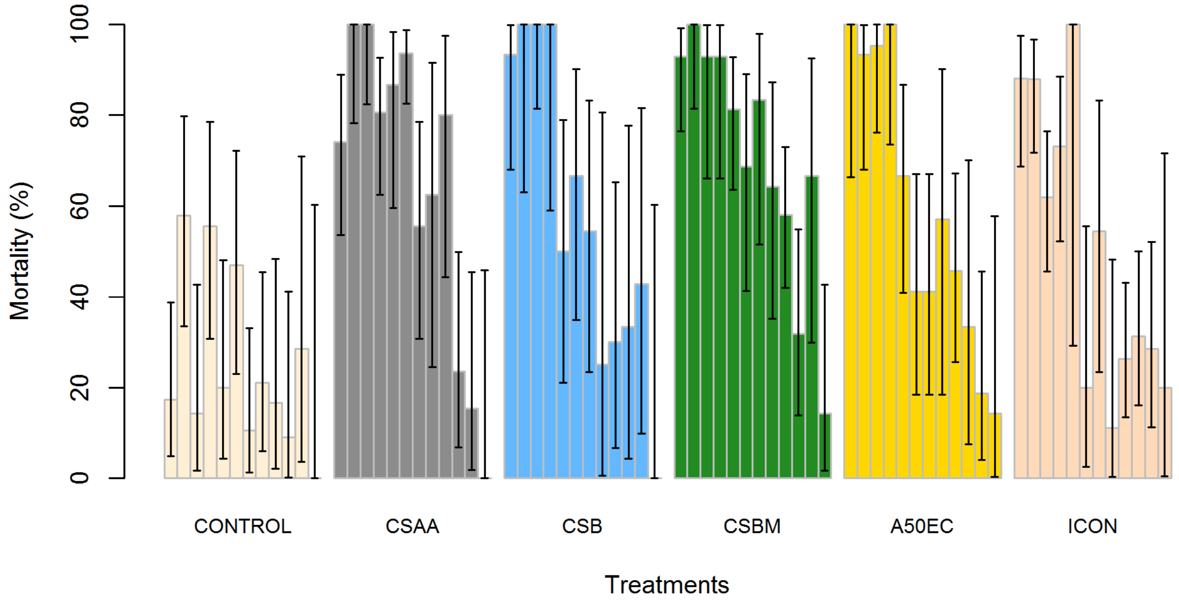

Figure 2.5. Crude mortality of anophelines other than *An. gambiae* or *An. funestus* in cement huts by month post treatment. See Figure 2.1 for legend.

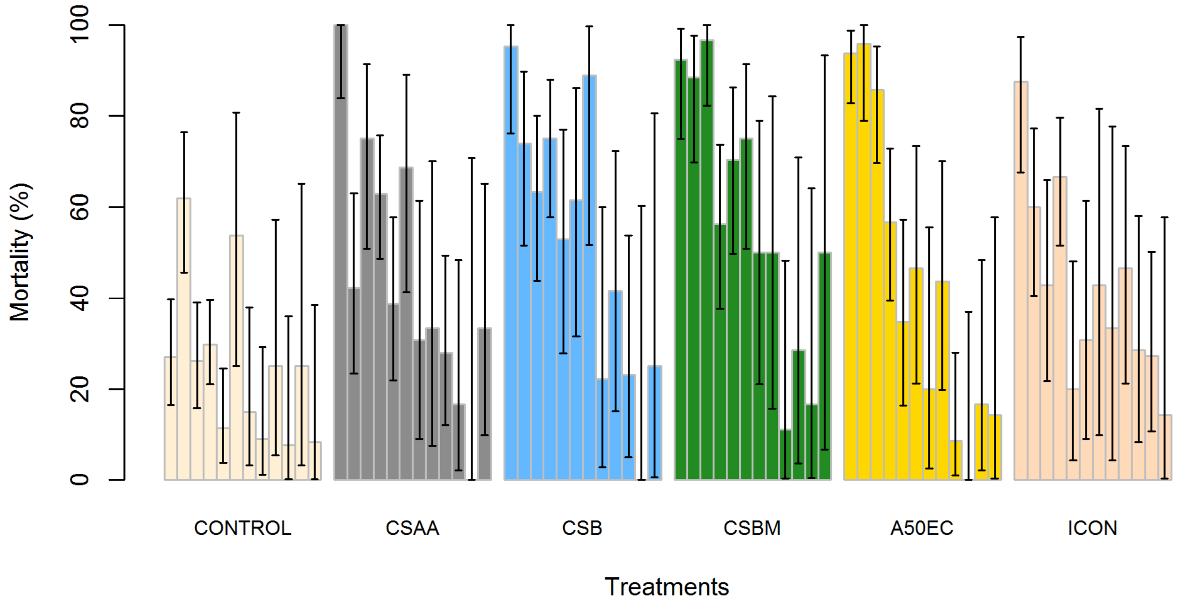

Figure 2.6. Crude mortality of anophelines other than *An. gambiae* or *An. funestus* in mud huts by month post treatment. See Figure 2.1 for legend.

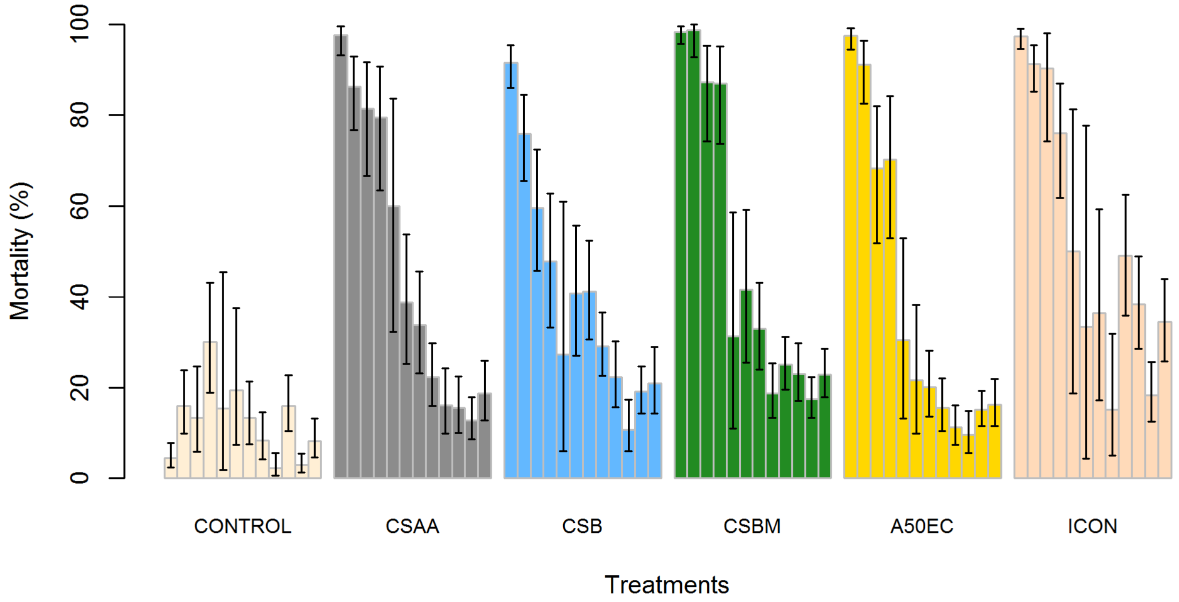

Mortality of non-anopheline genera in cement huts

Figure 2.7. Crude mortality of non-anopheline taxa in cement huts by month post treatment. See Figure 2.1 for legend.

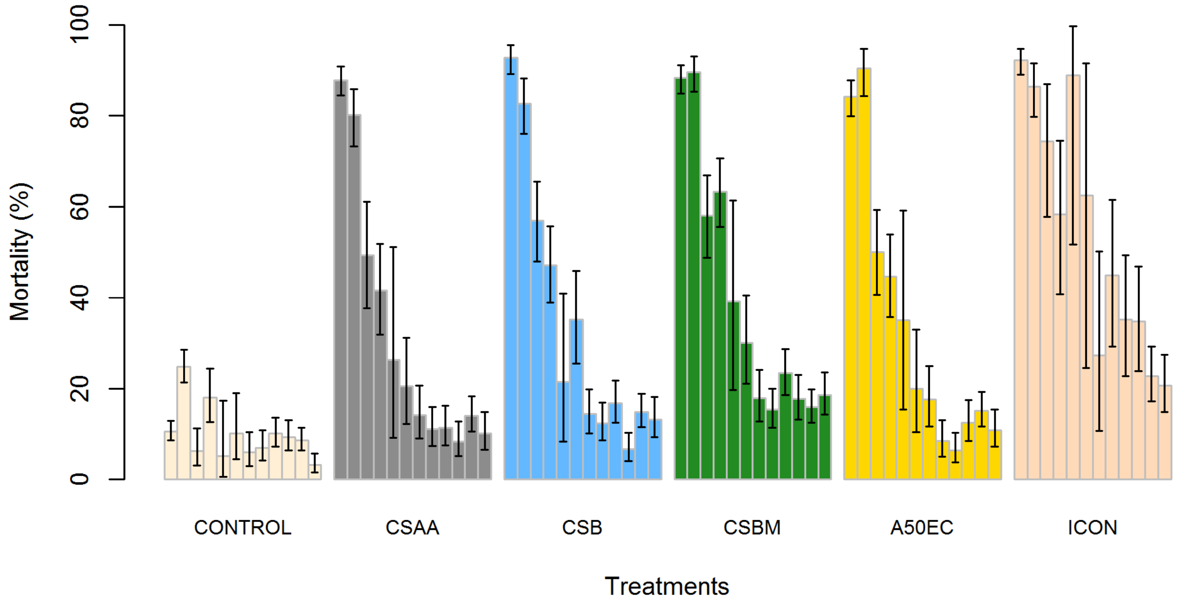

Figure 2.7. Crude mortality of non-anopheline taxa in mud huts by month post treatment. See Figure 2.1 for legend.
