# Supplementary material for: Micro-encapsulated pirimiphos-methyl shows high insecticidal efficacy and long residual activity against pyrethroid-resistant malaria vectors in central Côte d’Ivoire
Source: Malar J. 2014 Aug 25;13:332. doi: 10.1186/1475-2875-13-332 (PMC4159530; doi:10.1186/1475-2875-13-332)
Supplement: Supplementary file 3 — Additional file 3: Total numbers of nightly mosquito catches in experimental huts. (PDF 115 KB) [file 12936_2014_3370_MOESM3_ESM.pdf]

Additional file 3: Total numbers of nightly mosquito catches in experimental huts.

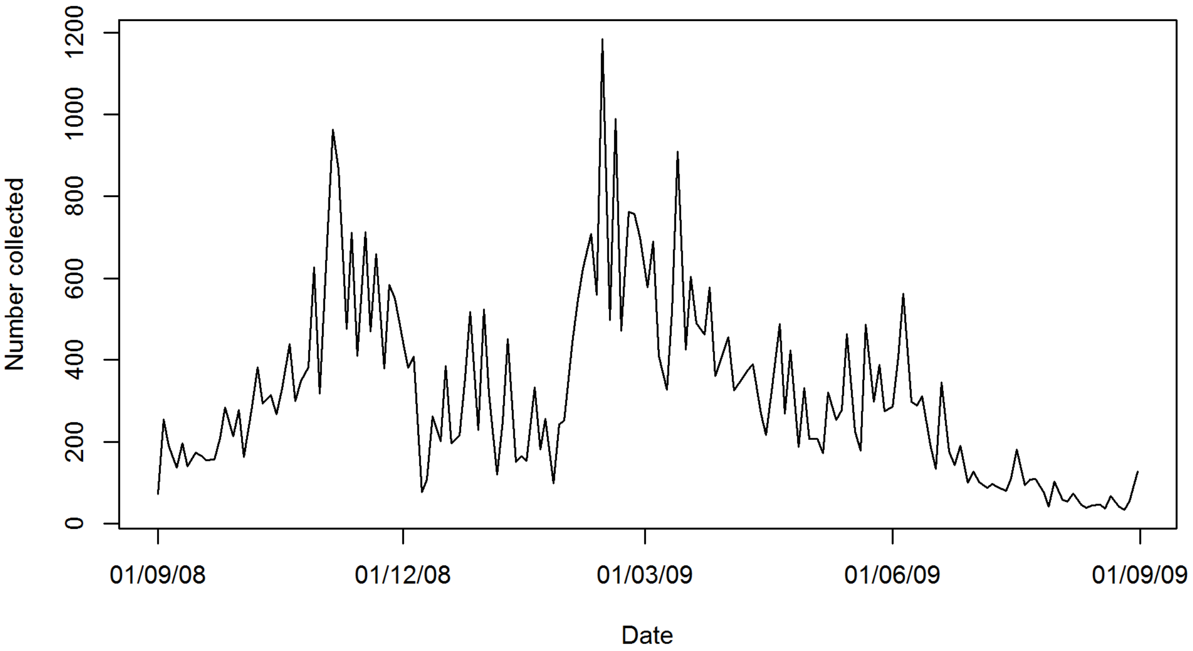

Figure 3.1. Total number of *An. gambiae* s.l. collected in all 24 experimental huts per trapping night.

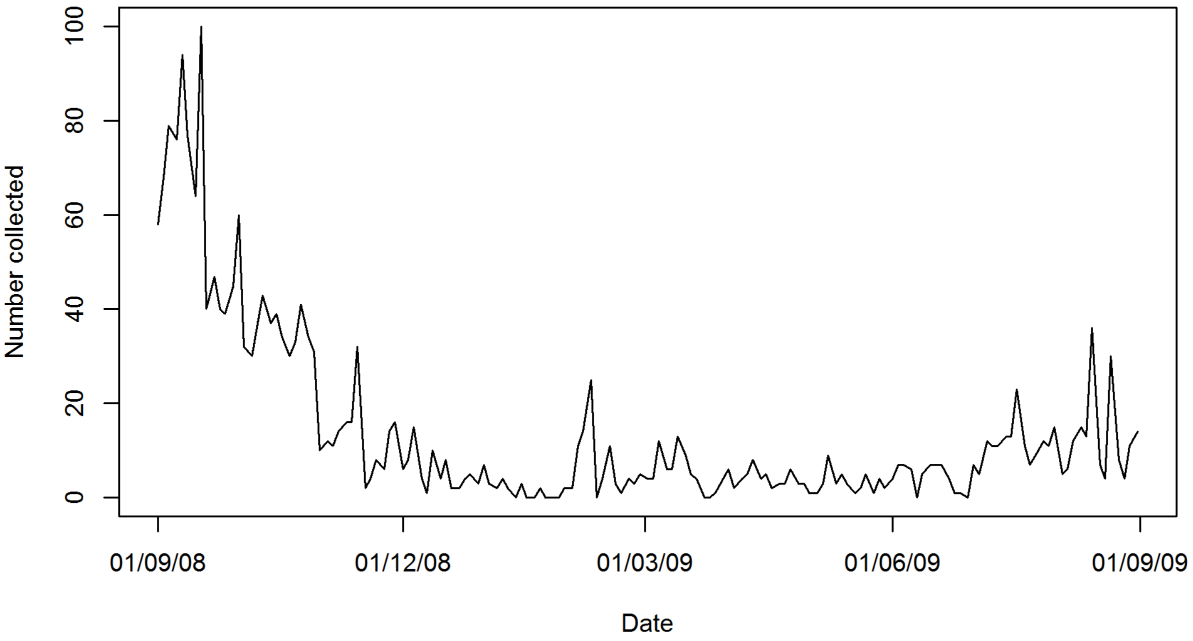

Figure 3.2. Total number of *An. funestus* collected in all 24 experimental huts per trapping night.

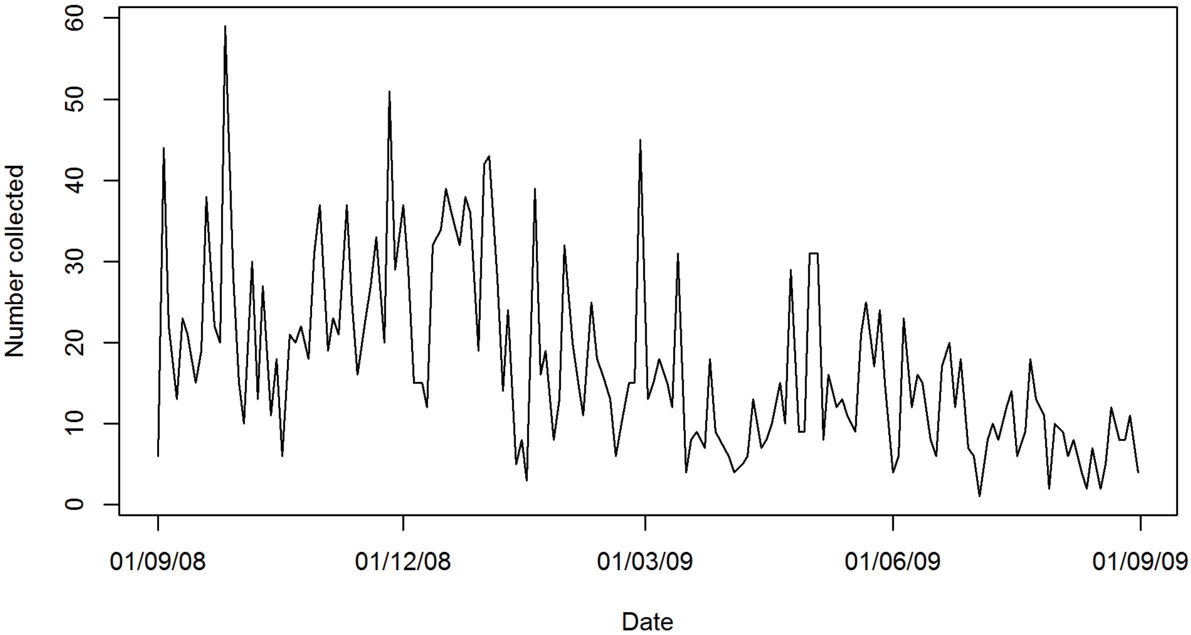

Figure 3.3. Total number of anophelines other than *An. gambiae* s.l. and *An. funestus* collected in all 24 experimental huts per trapping night.

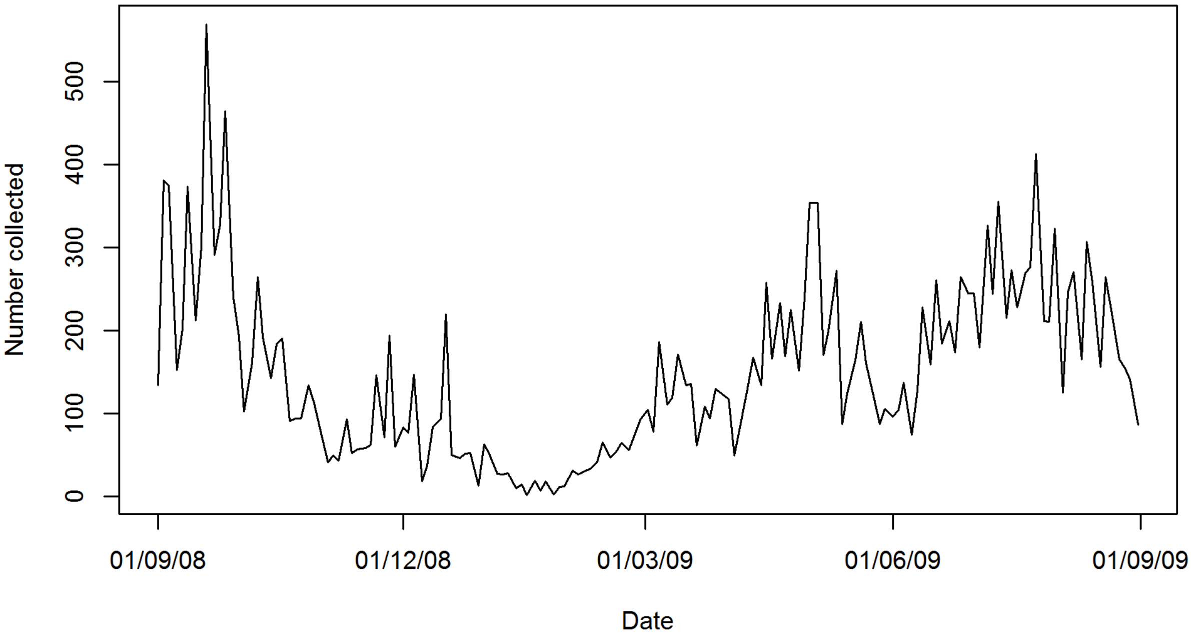

Figure 3.4. Total number of mosquito species other than anophelines collected in all 24 experimental huts per trapping night.
