# Supplementary material for: Micro-encapsulated pirimiphos-methyl shows high insecticidal efficacy and long residual activity against pyrethroid-resistant malaria vectors in central Côte d’Ivoire
Source: Malar J. 2014 Aug 25;13:332. doi: 10.1186/1475-2875-13-332 (PMC4159530; doi:10.1186/1475-2875-13-332)

Additional file 6. Crude results from experimental hut trials of pirimiphos-methyl and lambda-cyhalothrin on *An. funestus* by hut

Legend: See Figure 2.

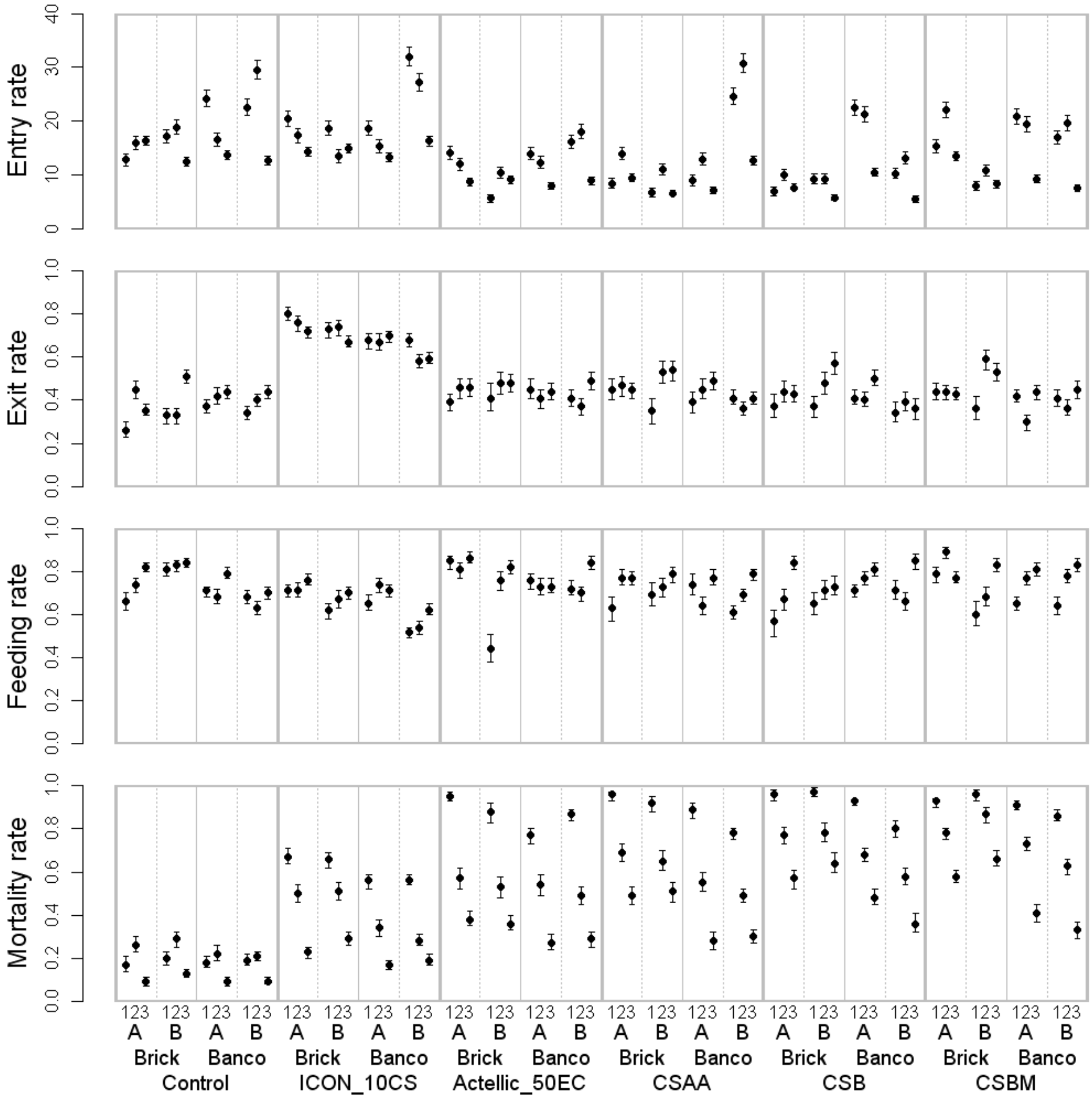

Supplement: Supplementary file 6 — Additional file 6: Crude results from experimental hut trials of pirimiphos-methyl and lambda-cyhalothrin on Anopheles funestus by hut. (PDF 37 KB) [file 12936_2014_3370_MOESM6_ESM.pdf]
