# Supplementary material for: Micro-encapsulated pirimiphos-methyl shows high insecticidal efficacy and long residual activity against pyrethroid-resistant malaria vectors in central Côte d’Ivoire
Source: Malar J. 2014 Aug 25;13:332. doi: 10.1186/1475-2875-13-332 (PMC4159530; doi:10.1186/1475-2875-13-332)

Additional file 7. Crude results from experimental hut trials of pirimiphos-methyl and lambda-cyhalothrin on other than *An. gambiae* s.l. and *An. funestus* by hut

Legend: See Figure 2.

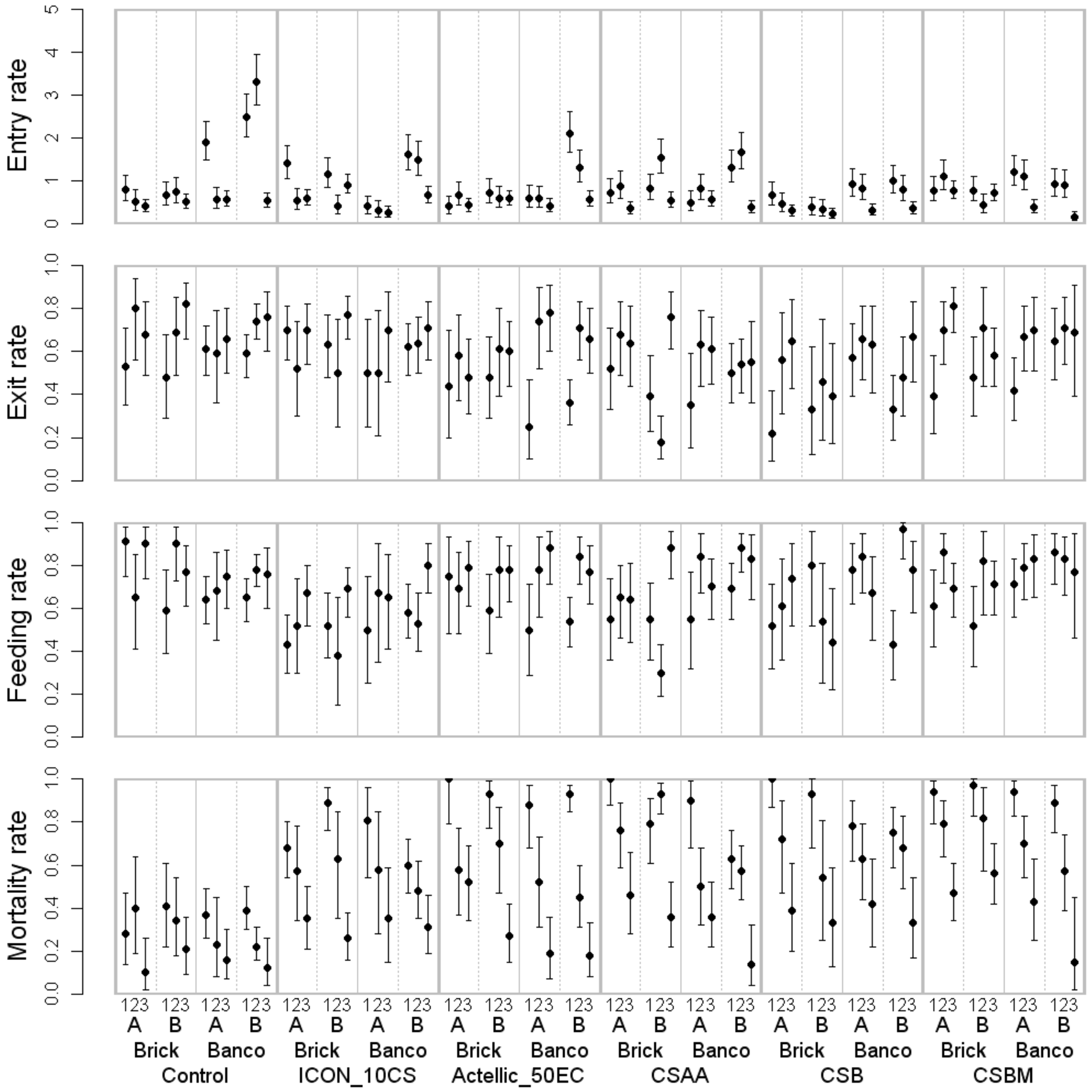

Supplement: Supplementary file 7 — Additional file 7: Crude results from experimental hut trials of pirimiphos-methyl and lambda-cyhalothrin on other than Anopheles gambiae s.l. and Anopheles funestus by hut. (PDF 40 KB) [file 12936_2014_3370_MOESM7_ESM.pdf]
