# Supplementary material for: Micro-encapsulated pirimiphos-methyl shows high insecticidal efficacy and long residual activity against pyrethroid-resistant malaria vectors in central Côte d’Ivoire
Source: Malar J. 2014 Aug 25;13:332. doi: 10.1186/1475-2875-13-332 (PMC4159530; doi:10.1186/1475-2875-13-332)

Additional file 9. Effects of pirimiphos-methyl and lambda-cyhalothrin on *An. funestus*
Legend: See Figure 3.

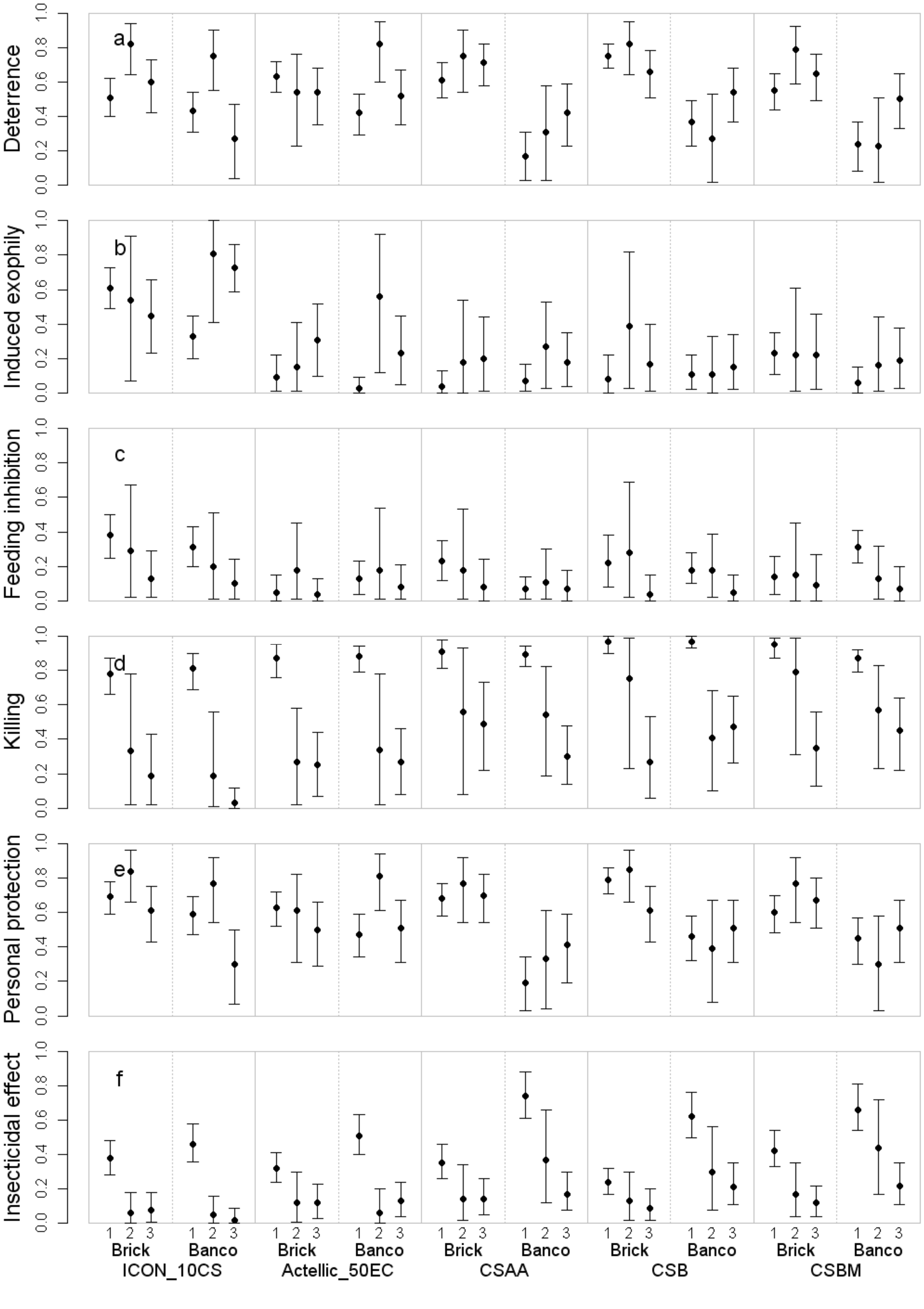

Supplement: Supplementary file 9 — Additional file 9: Effects of pirimiphos-methyl and lambda-cyhalothrin on Anopheles funestus . (PDF 40 KB) [file 12936_2014_3370_MOESM9_ESM.pdf]
