# Supplementary material for: Micro-encapsulated pirimiphos-methyl shows high insecticidal efficacy and long residual activity against pyrethroid-resistant malaria vectors in central Côte d’Ivoire
Source: Malar J. 2014 Aug 25;13:332. doi: 10.1186/1475-2875-13-332 (PMC4159530; doi:10.1186/1475-2875-13-332)

Additional file 11. Odds ratio of overall insecticidal effect of pirimiphos-methyl relative to lambda-cyhalothrin on *An. funestus*

Legend: See Figure 4.

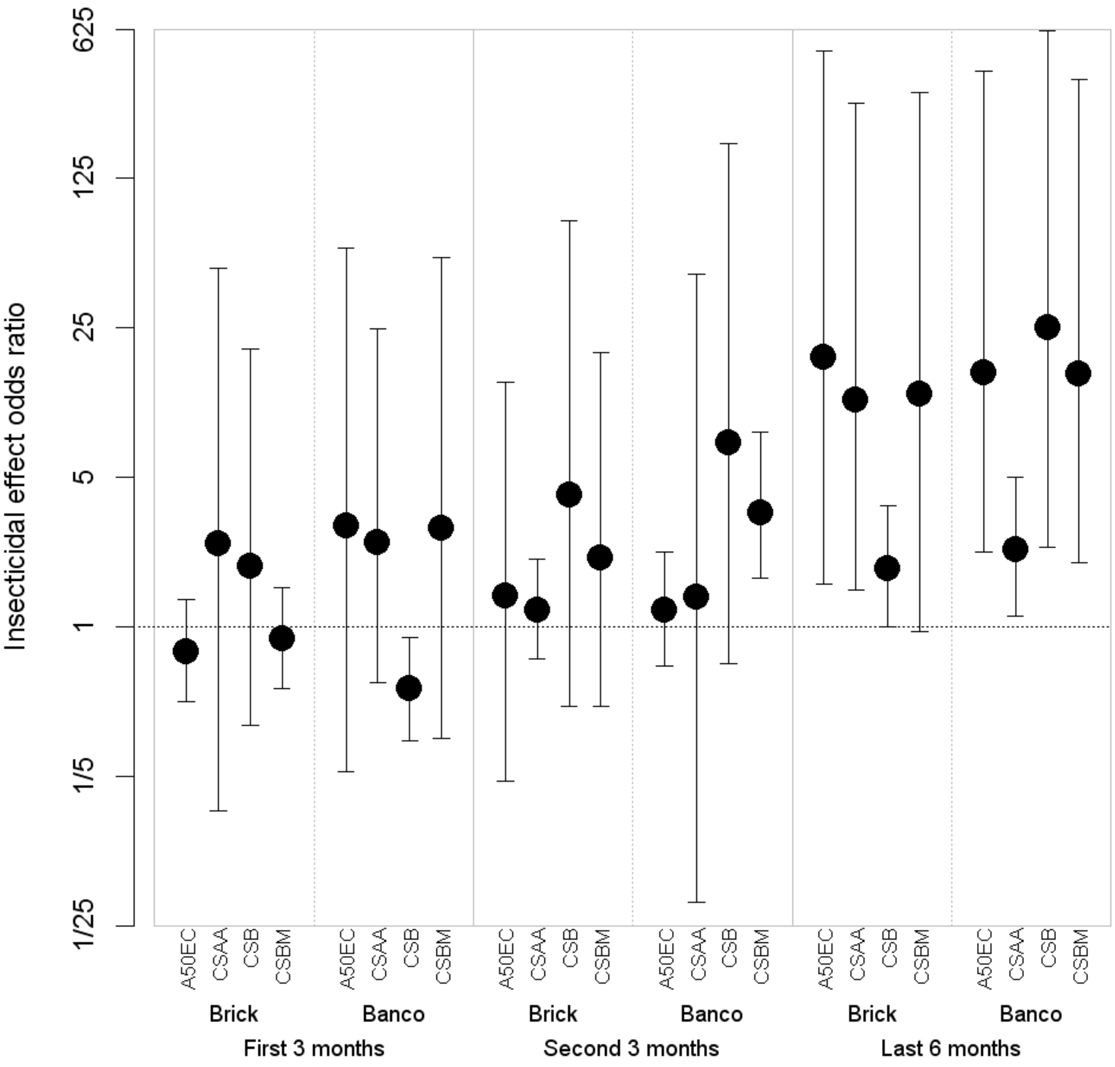

Supplement: Supplementary file 11 — Additional file 11: Odds ratio of overall insecticidal effect of pirimiphos-methyl relative to lambda-cyhalothrin on Anopheles funestus . (PDF 28 KB) [file 12936_2014_3370_MOESM11_ESM.pdf]
