# Supplementary material for: Micro-encapsulated pirimiphos-methyl shows high insecticidal efficacy and long residual activity against pyrethroid-resistant malaria vectors in central Côte d’Ivoire
Source: Malar J. 2014 Aug 25;13:332. doi: 10.1186/1475-2875-13-332 (PMC4159530; doi:10.1186/1475-2875-13-332)

Additional file 12. Odds ratio of overall insecticidal effect of pirimiphos-methyl relative to lambda-cyhalothrin on on anophelines other than *An. gambiae* s.l. and *An. funestus*
Legend: See Figure 4.

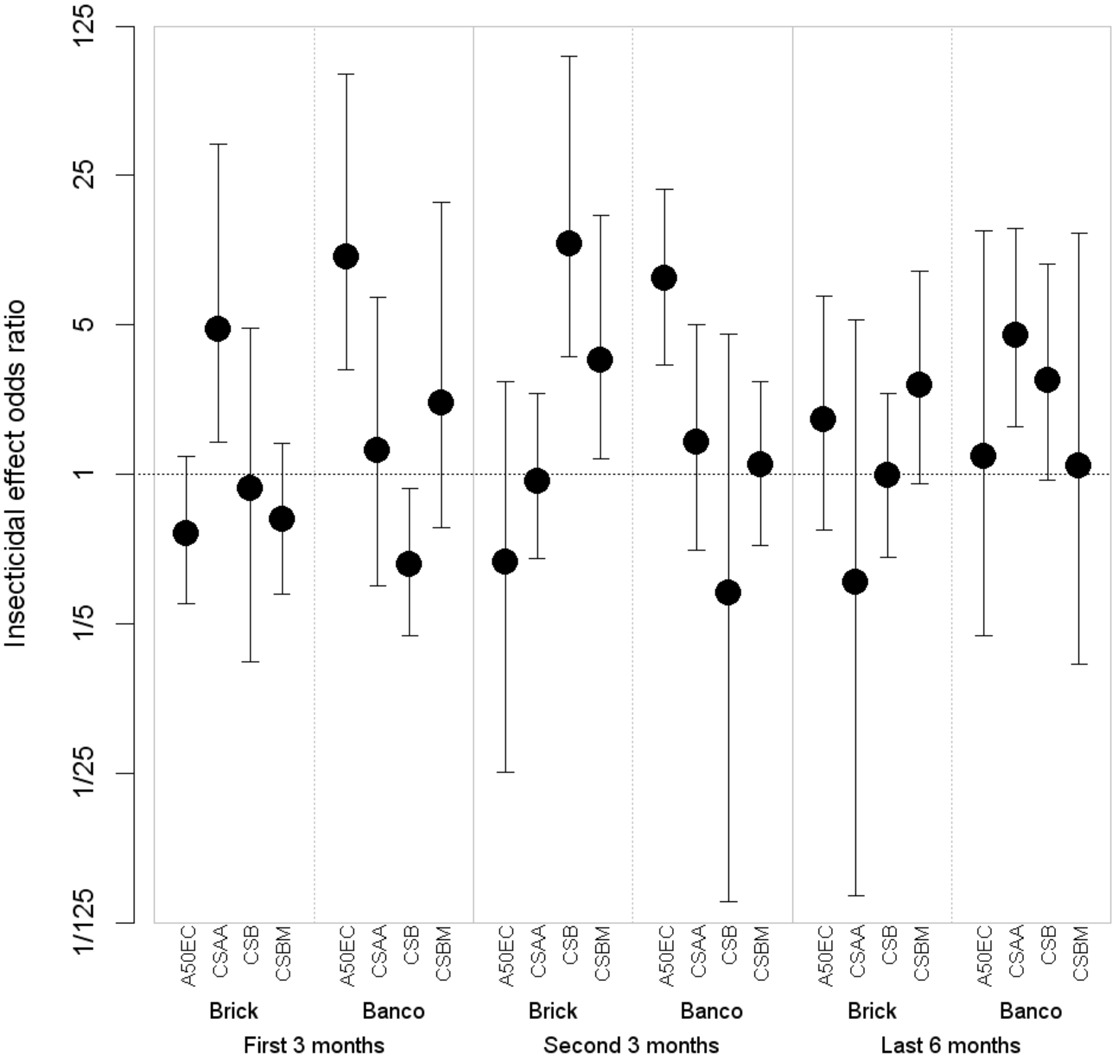

Supplement: Supplementary file 12 — Additional file 12: Odds ratio of overall insecticidal effect of pirimiphos-methyl relative to lambda-cyhalothrin on on anophelines other than Anopheles gambiae s.l. and Anopheles funestus . (PDF 29 KB) [file 12936_2014_3370_MOESM12_ESM.pdf]
