# Supplementary material for: Micro-encapsulated pirimiphos-methyl shows high insecticidal efficacy and long residual activity against pyrethroid-resistant malaria vectors in central Côte d’Ivoire
Source: Malar J. 2014 Aug 25;13:332. doi: 10.1186/1475-2875-13-332 (PMC4159530; doi:10.1186/1475-2875-13-332)
Supplement: Supplementary file 13 — Additional file 13: Perceived strengths and weakness of different insecticide formulations in experimental huts. (PDF 15 KB) [file 12936_2014_3370_MOESM13_ESM.pdf]

Additional file 13: Perceived strengths and weakness of different insecticide formulations in experimental huts.

Product	Wall type	Odor (1=very strong, 7=very weak)			Efficacy (1= very strong, 7=very weak)			Pleasant (1= very pleasant, 7=not pleasant)			Not dangerous (1= very dangerous, 7=not dangerous)			Used to (1= easily get used to, 7= trouble getting used to)		
		Mean	95% Conf. Int.		Mean	95% Conf. Int.		Mean	95% Conf. Int.		Mean	95% Conf. Int.		Mean	95% Conf. Int.	
**Untreated**	Mud	5.43	4.76	6.10	4.90	4.07	5.73	6.18	5.58	6.78	4.28	3.40	5.15	4.50	3.67	5.33
	Cement	5.89	4.89	6.90	6.05	5.23	6.88	5.57	3.98	7.16	3.00	1.86	4.14	3.26	2.01	4.52
**CS B**	Mud	5.61*	5.44	5.78	5.49 *	5.31	5.67	5.35	5.18	5.53	3.86	3.68	4.04	3.89	3.71	4.07
	Cement	5.05	4.81	5.28	5.06	4.82	5.30	4.48	4.18	4.79	2.63	2.41	2.85	2.77	2.54	3.00
**ICON 10 CS**	Mud	5.81	5.65	5.97	5.69	5.52	5.86	5.44	5.26	5.62	3.79	3.60	3.98	3.90	3.71	4.09
	Cement	5.16	4.92	5.40	5.19	4.95	5.43	4.22	3.90	4.53	2.86	2.62	3.10	2.84	2.60	3.08
**CS BM**	Mud	5.81	5.65	5.97	5.67	5.50	5.84	5.29	5.10	5.48	3.80	3.61	3.99	3.82	3.64	4.01
	Cement	4.76**	4.50	5.02	4.76**	4.50	5.02	3.98	3.65	4.31	2.55	2.33	2.78	2.52	2.29	2.75
**Actellic 50 EC**	Mud	5.75	5.59	5.92	5.61	5.44	5.79	5.24	5.04	5.44	3.73	3.54	3.92	3.74	3.56	3.93
	Cement	5.29	5.05	5.52	5.24	5.00	5.48	3.96	3.65	4.27	2.78	2.54	3.01	2.74	2.51	2.98
**CS AA**	Mud	5.81	5.66	5.97	5.59	5.41	5.77	5.29 *	5.09	5.48	3.73*	3.54	3.92	3.68*	3.49	3.87
	Cement	5.70	5.49	5.91	5.59	5.37	5.81	3.85**	3.51	4.19	2.28**	2.07	2.49	2.36**	2.15	2.58

* Best value for 'Mud'.

** Best value for 'Cement'
