# Supplementary material for: Micro-encapsulated pirimiphos-methyl shows high insecticidal efficacy and long residual activity against pyrethroid-resistant malaria vectors in central Côte d’Ivoire
Source: Malar J. 2014 Aug 25;13:332. doi: 10.1186/1475-2875-13-332 (PMC4159530; doi:10.1186/1475-2875-13-332)
Supplement: Supplementary file 14 — Additional file 14: Results of WHO cone tests with wild An. gambiae s.l. from Yaokoffikro. (PDF 70 KB) [file 12936_2014_3370_MOESM14_ESM.pdf]

Additional file 14: Results of WHO cone tests with wild *An. gambiae* s.l. from Yaokoffikro

Wild *An. gambiae* s.l. adults were obtained from larval collections at natural breeding sites in Yaokoffikro (38 km south of the M'Bé station). Larvae were transferred to a laboratory at the Institut Pierre Richet (IPR) in Bouaké for rearing to adults, and provided with a diet of Tetra Mikromin fish food until adult emergence. WHO susceptibility tests were performed on 2- to 5-day -old unfed wild-caught females reared from larval collections, using standard WHO test kits and protocols for adult mosquitoes. In brief, papers impregnated with 0.05% deltamethrin, 0.75% permethrin, and 4% DDT were sourced from WHO. Batches of 20 – 25 females were exposed to impregnated papers in WHO test tubes for 1 hour with at least four replicates per bioassay and concurrent negative controls with corresponding insecticide-free papers. After 60 min mosquitoes were transferred to holding containers with access to a 10% honey solution. Results are shown in Figure 14.1.

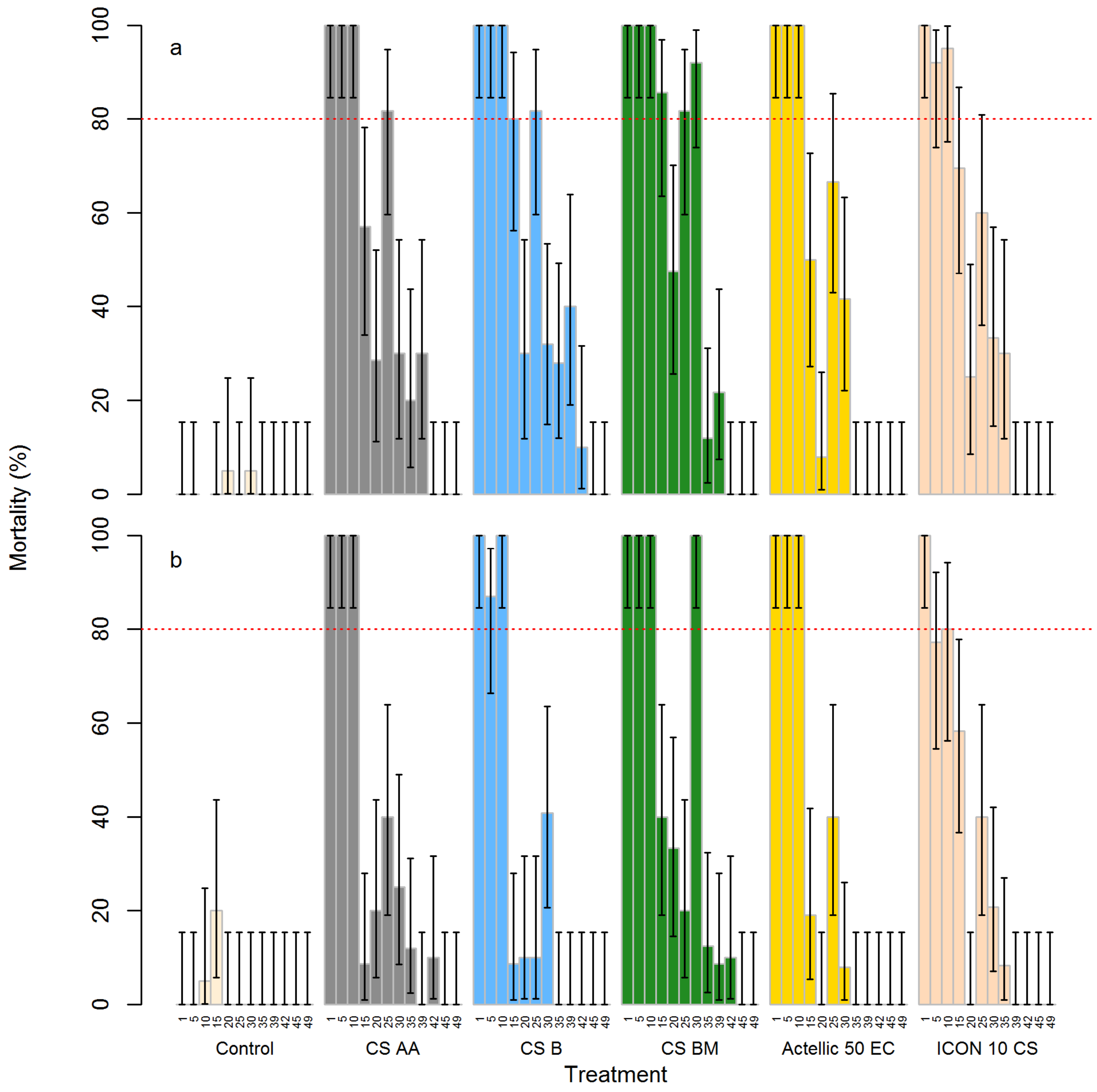

Figure 14.1. Residual activity of insecticide formulations against wild *Anopheles gambiae*. Mortality in WHO cone tests after 24 hours of *An. gambiae* s.l. collected as larvae in Yaokoffikro and raised to adults in the insectary **a** on cement surfaces, and **b** on mud surfaces. Error bars show approximate 95% confidence intervals (data on the exact number of mosquitoes tested was lost and only the per cent mortality was recorded). The red dotted line shows the WHO threshold of 80%. The first horizontal axis labels refer to the week of testing post treatment.
